# TNF-α and IFN-γ impair neural oscillations and induce neurodegeneration by microglial nitric oxide, metabolic and oxidative stress

**DOI:** 10.1186/s12974-026-03835-x

**Published:** 2026-05-08

**Authors:** Nikolai Malorny, Bruno Chausse, Babak Khodaie, Amr Elgez, Lennart Söder, Andrea Lewen, Alexei V. Egorov, Oliver Kann

**Affiliations:** 1 Institute of Physiology and Pathophysiology, Heidelberg University, Im Neuenheimer Feld 326, D-69120 Heidelberg, Germany; 2 Interdisciplinary Center for Neurosciences (IZN), Heidelberg University, D-69120 Heidelberg, Germany

**Keywords:** Action potential, Cytokines, Electrophysiology, Energy metabolism, Gamma oscillations, Microglial cells, Neuroinflammation, Neuronal cell death, Reactive oxygen and nitrogen species (ROS/RNS)

## Abstract

**Background:**

The cytokine tumor necrosis factor-alpha (TNF-α) regulates inflammatory responses in infectious and neurodegenerative diseases and also affects neuronal function. The role of TNF-α in the activation of microglial cells (resident central nervous system macrophages), including the impact on neuronal survival, excitability, and synaptic transmission is incompletely defined, however. We explored the effects of chronic TNF-α exposure (72 h) on microglia and neurons in organotypic hippocampal slice cultures from male and female rats, i.e., postnatal cortex tissue lacking leukocyte invasion and adaptive immunity.

**Methods:**

We applied gene expression analysis, biochemical assays, immunohistochemistry, electrophysiology by extracellular (local field potential) and intracellular (intrinsic membrane properties) recordings, and pharmacological ablation of the microglial cell population. We mainly focused on carbachol-induced neural network oscillations (brain waves) in the gamma frequency band (30–70 Hz) that underlie higher cognitive functions such as perception, attention, and memory.

**Results:**

TNF-α induced microglial proliferation and upregulation of genes related to inflammation and oxidative stress such as *Il6* (interleukin-6), *Nos2* [inducible nitric oxide (NO) synthase, iNOS] and *Sod2* (superoxide dismutase 2), which was accompanied by a decreased number of slices showing gamma oscillations in extracellular recordings. Notably, a fraction of slices presented neural bursting reflecting hyperexcitability in the tissue. Neuronal dysfunction was absent during acute TNF-α exposure (30 min). When paired with the lymphocyte cytokine interferon-gamma (IFN-γ), TNF-α induced an amplified neuroinflammation response dominated by bursting or loss of electrical activity. In intracellular recordings, neurons showed a brief burst of action potentials followed by slowing of spiking with pronounced afterhyperpolarization (switch from regular to burst firing behavior) during depolarizing current injection. Notably, the impairments could be attenuated by inhibition of iNOS and NADPH oxidase, glucose supplementation, microglial depletion or blockade of TNF receptor 1 (TNFR1) signaling with small molecule drugs, RIPA-56 and ICCB-19.

**Conclusions:**

Our data provide mechanistic insight into TNF-α- and IFN-γ-induced neuronal impairments mediated by microglial NO, metabolic and oxidative stress, and demonstrate functional neuroprotection by pharmacology. Our study extends the pathophysiological understanding of diseases such as sepsis, multiple sclerosis, Alzheimer’s disease, depression and schizophrenia featuring activated microglia, infiltrating monocytes and T cells, and/or blood-brain barrier leakage.

**Graphical Abstract:**

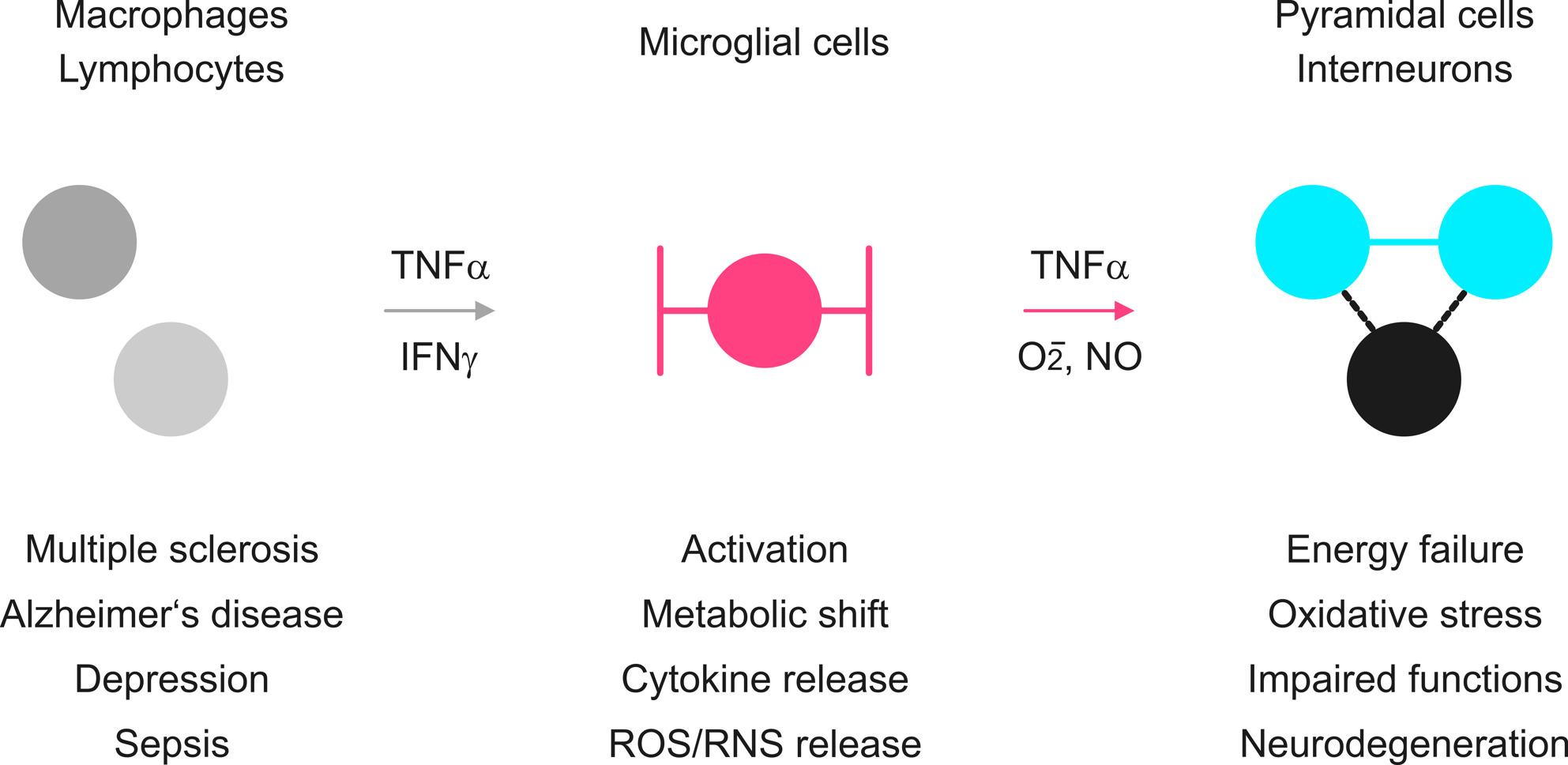

**Supplementary Information:**

The online version contains supplementary material available at 10.1186/s12974-026-03835-x.

## Introduction

Tumor necrosis factor-alpha (TNF-α) is a pleiotropic cytokine involved in induction of inflammation, defense against pathogens and support of tissue regeneration in various organs, including the brain [1–3]. TNF-α can additionally affect neuronal synaptic transmission, cognition, and behavior [4–8].

In the central nervous system (CNS), TNF-α is mainly released by activated microglial cells (resident macrophages) that also feature proliferation, migration, release of cytokines and chemokines, production of reactive oxygen and nitrogen species (ROS/RNS) and phagocytosis in pathological conditions [1, 9–12]. In addition, T lymphocytes (T cells), natural killer cells and monocytes infiltrating the brain parenchyma in pathologic conditions as well as various systemic infections can contribute to elevated TNF-α levels [1–3, 13].

TNF-α mainly activates the TNF receptor (TNFR) 1 that is constitutively expressed in most nucleated cells, including microglia, neurons and astrocytes [12, 14]. The TNFR2 is more restricted to immune and endothelial cells and preferentially activated by membrane-bound TNF-α [15, 16]. In the brain, TNFR1 signaling associates with facilitation of epileptic seizure onset and potentiation of excitotoxicity, which is currently thought to mainly result from increased expression of excitatory glutamatergic AMPA receptors, enhanced endocytosis of inhibitory GABA_A_ receptors, and, perhaps, enhanced glutamate release from presynaptic terminals [7, 8, 12, 17]. However, TNFR1 signals through various intracellular transduction pathways and induces complex inflammatory responses, including programmed cell death [15, 18, 19]. Notably, the early studies on TNF-α-mediated ROS/RNS production in the brain were controversial and did not address the role of activated microglia in detail [20–23], and a later transcription profiling study was done in monoculture of primary rat microglia not addressing functional microglia-neuron interactions [24].

Interferon-gamma (IFN-γ) is another key cytokine mainly released by activated T lymphocytes and natural killer cells [25]. IFN-γ is important for inhibition of viral replication and suppression of tumor cells. In addition, IFN-γ induces proinflammatory and microbicidal responses by priming of tissue macrophages, including microglia [3, 10, 25]. In rat hippocampal slice cultures, for example, single exposure to IFN-γ induces microglial proliferation and nitric oxide (NO) release that moderately affects synaptic transmission and neural network oscillations, notably, without neurodegeneration [26, 27].

TNF-α and IFN-γ have been implicated in the pathogenesis of various CNS diseases, including epilepsy, neuropathic pain, multiple sclerosis, Alzheimer’s disease and depression [10–12, 14, 18, 28]. Although crucial for the clinical understanding of cognitive impairment in neurologic and psychiatric patients, the roles of TNF-α and IFN-γ in microglial activation and inflammatory neuronal disturbances are still incompletely defined [12, 13, 29–33]. We hypothesized that TNF-α and IFN-γ induce microglial ROS/RNS production and thus contribute to neuronal dysfunction and neurodegeneration.

We used rat hippocampal slice cultures that permit acute and chronic exposures of cortical tissue to various inflammatory ligands like exogenous lipopolysaccharide (LPS) and endogenous IFN-γ, in the absence of infiltrating leukocytes [26, 27, 34, 35]. In slice cultures, the cytoarchitecture is well preserved and natural synaptic transmission, which is reflected by neural network oscillations in the gamma frequency band (30–70 Hz) underlying higher brain functions in vivo, can be reliably induced [26, 27, 36]. Notably, impairment of gamma oscillations provides a sensitive readout of metabolic and oxidative stress in neuronal networks thus permitting studies in functional neuroimmunology [26, 37–39].

In essence, we demonstrate that TNF-α and IFN-γ impair neural oscillations and induce neurodegeneration by a microglial NO mechanism that is sensitive to metabolic support and pharmacological intervention.

## Materials and methods

### Rats

Male and female Wistar rats (Janvier Laboratories, Le Genest-Saint-Isle, France) were used for experiments performed and reported in accordance with the ARRIVE 2.0 guidelines. Handling and experiments were in accordance with the European directive 2010/63/EU and approved by the animal welfare office at Heidelberg University (licenses T-45/18, T-37/21 and T-27/24). For most experiments, slice cultures were prepared from male pups. For some key experiments, slice cultures were prepared from female pups to explore possible differences due to sex-related variability on neuronal and microglial biology [40].

### Slice cultures

Organotypic hippocampal slice cultures were prepared as previously described [26, 36]. Nine days old Wistar rats were killed by decapitation. The hippocampi were quickly removed, cooled to about 4 °C in minimal essential medium saturated with 95% O_2_ plus 5% CO_2_ (vol/vol) and then cut into slices of 400 μm thickness using a McIlwain tissue chopper (Mickle Laboratory Engineering Company Ltd., Guildford, UK) under sterile conditions. Three slices with intact hippocampal structures were placed on Biopore^®^ membrane inserts (Millicell standing inserts, Merck Millipore, Darmstadt, Germany) and kept in a Heracell^®^ incubator (Thermo-Fisher Scientific, Dreieich, Germany) at the interface between culture medium [50% minimal essential medium, 25% Hank’s balanced salt solution (Sigma-Aldrich, Taufkirchen, Germany), 25% heat-inactivated horse serum (Life Technologies, Darmstadt, Germany), additionally 2 mM L-glutamine (Life Technologies), titrated to pH 7.3 with Trisbase] in humidified normal atmosphere enriched with 5% (vol/vol) CO_2_ at 36.5 °C. The glucose concentration in the culture medium was about 4 mM [36, 41]. Culture medium was exchanged three times per week until day in vitro (DIV) 9. Culture medium was free of antibiotics that are known to potentially affect mitochondrial function in various cell types [42, 43].

### Exposures and microglial ablation

Biopore^®^ membranes with slice cultures were randomly assigned to experimental groups. Slice cultures were exposed at DIV 9 to recombinant rat TNF-α (1-1000 ng/mL) or TNF-α (100 ng/mL) plus IFN-γ (100 ng/mL) (both PeproTech, Hamburg, Germany) for 72 h (referred to as chronic exposure). Microglia were depleted chemically from DIV 0 on by continuously adding liposome-encapsulated clodronate (100 µg/mL) (Liposoma B.V., Amsterdam, The Netherlands) to the culture medium [26, 36, 44]. Apocynin (100 µM), and *N*-3-aminomethyl-benzyl-acetamidine (1400W) (400 µM), RIPA-56 (50 µM or 100 µM) and ICCB-19 (50 µM) (all from Sigma-Aldrich, Taufkirchen, Germany) were added to the medium at DIV 9. Exposures were performed in the dark and culture medium was not exchanged during exposure from DIV 9 to 12. Exposures from a given animal always included at least one control and one positive control (TNF-α and/or TNF-α plus IFN-γ) along with other drug treatments.

### Biochemical assays

Each sample of culture medium was collected from one Biopore^®^ membrane carrying three slice cultures and frozen to -80 °C. Clodronate-containing medium was centrifuged prior to freezing. Enzyme-linked immunosorbent assay kits were purchased from R&D Systems (Minneapolis, MN, USA; via Bio-Techne, Wiesbaden, Germany), and analysis was performed following the manufacturer’s protocol for the detection of interleukin (IL) 6 (IL-6, Cat. num. DY506). In brief, a 96-well plate was coated with capture antibody diluted in phosphate buffered saline (PBS) (pH 7.2–7.4) overnight. Detection antibody was diluted in reagent diluent buffer with 2% normal goat serum (NGS). Seven-point standard curves were constructed by twofold dilution steps of 8000 pg/mL recombinant IL-6. Detection antibody was applied for 2 h and tetramethylbenzidine substrate (Moss Inc., Pasadena, USA) was used for color development. Optical density was measured with a microplate reader (iMark, Bio-Rad GmbH, Munich, Germany) at 450 nm (with 540 nm reference) after stopping the reaction with sulfuric acid. IL-6 concentrations were determined by using a linear or quadratic fit. NO release was analyzed by measuring the stable metabolite nitrite with a Griess reaction used on undiluted culture medium. Nine-point standard curves were constructed by twofold dilution steps of 80 µM sodium nitrite high standard (Merck Chemicals, Darmstadt, Germany). After adding the Griess reagent mixture (0.05% 1-naphthylethylenediamine hydrochloride, 0.5% sulfanilamide and 2.5% orthophosphoric acid), the optical density was measured with a microplate reader (Bio-Rad) at 540 nm. Nitrite concentrations were determined by using a linear fit.

### LDH activity

Lactate dehydrogenase (LDH) activity was assessed using an assay kit (Cat. num. MAK066, Sigma Aldrich) by following the manufacturer’s instructions. A master reaction mix containing LDH assay buffer and LDH substrate mix was used on samples, positive control and sole culture medium. A five-point standard curve (2.5–12.5 nmol/well) was used to derive NADH concentrations [42]. Reactions took place at 36 °C. Optical density was measured at 450 nm in one-minute steps for 20 min. Optical density of sole culture medium was subtracted from measurements. Enzyme activity was calculated using the following formula: LDH activity = NADH generation (nmol) * [reaction time (min) * volume (mL)]^−1^.

### RNA isolation and qRT-PCR

**Fig. 1:**
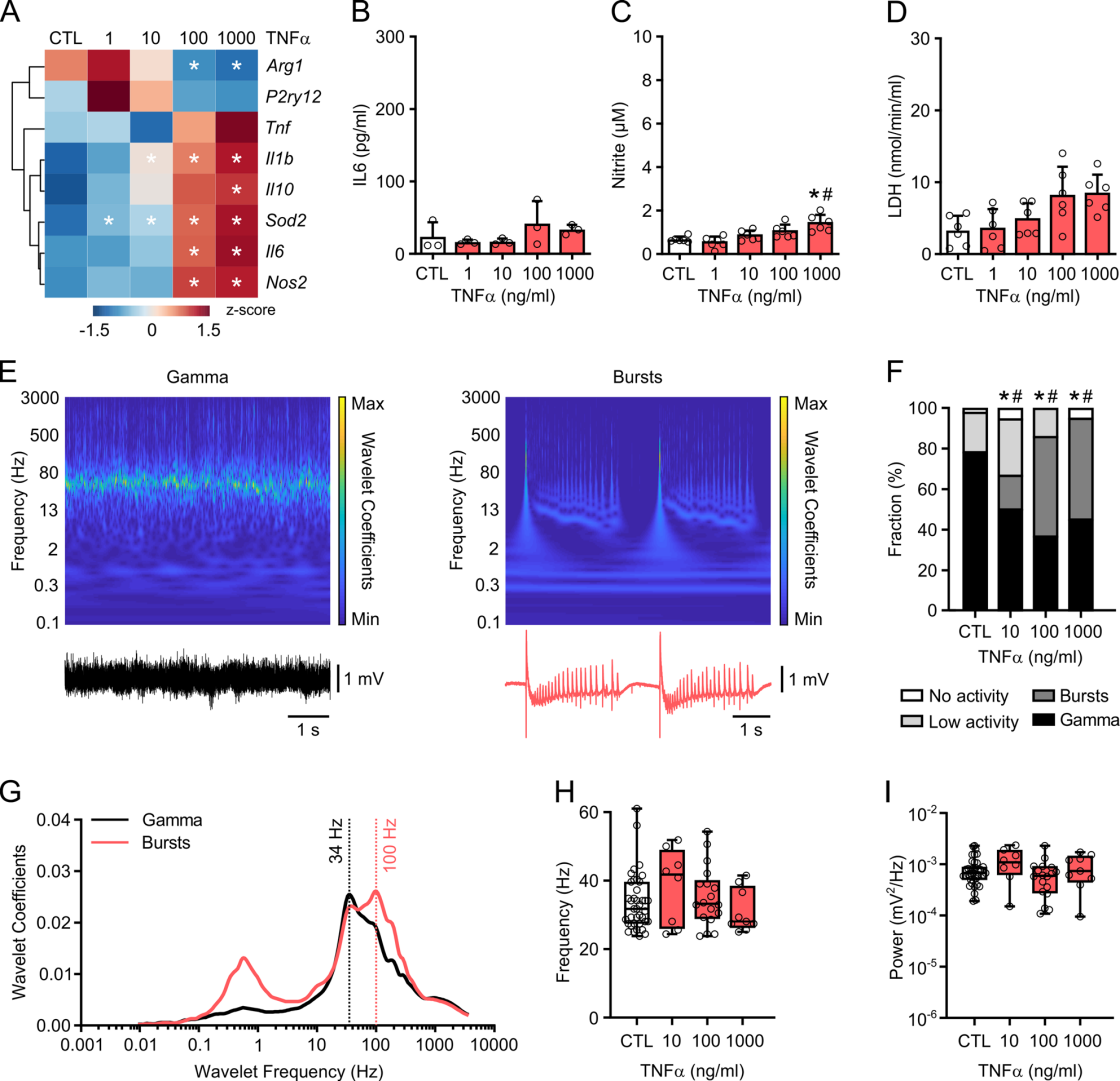
Chronic TNF-α induces neural bursting. Slice cultures were exposed to different concentrations of TNF-α (1-1000 ng/mL) for 72 h. Untreated slice cultures served as control (CTL). LFP recordings were made in the presence of carbachol (10 µM). **A** Heat-map depicting alterations in the expression of microglial and inflammatory genes for arginase 1, purinergic receptor P2Y12, TNF-α, IL-1β, IL-10, superoxide dismutase 2, IL-6, and nitric oxide (NO) synthase 2. Euclidean distance and Ward’s linkage were used for hierarchical clustering. Comparison by one-way ANOVA with Tukey’s post hoc test. *P < 0.05 vs. CTL. **B** IL-6 release, **C** content of the NO reaction product nitrite and **D** LDH activity in the culture medium. Each medium sample originated from three slice cultures. Comparison by one-way ANOVA with Tukey’s post hoc test (**B**) or Kruskal-Wallis test with Dunn’s post hoc test (**C**, **D**). *P < 0.011 vs. CTL, #*P* < 0.003 vs. TNFα 1 ng/mL. **E** Morlet wavelet spectra (top) calculated from gamma oscillations (Gamma) and neural bursting (Bursts) in LFP recordings (bottom). Magnitude values in Morlet wavelet spectra were normalized to the maximal value in each activity state. The scales are thus not comparable. **F** Distribution of network activities. Comparison by Fisher’s exact test. *P < 0.035 vs. CTL for Gamma vs. No Gamma. #*P* < 0.016 vs. CTL for Burst vs. No Burst. **G** Morlet wavelet magnitude spectra measured at the timepoints of bursts compared with gamma oscillations in TNFα. **H** Peak frequency and **I** peak power of slices showing gamma oscillations. Comparison by Kruskal-Wallis test with Dunn’s post hoc test. For n/N membranes/animals: (**A**) 3/3, (**B**) 3/3, (**C**) 6/6, (**D**) 6/6. For n/N slices/animals: (**F**, **H**, **I**) 18–51/6–21, (**G**) 13–17/10–12

Three slice cultures from one Biopore^®^ membrane were pooled as a single sample for RNA isolation. RNA was isolated with the RNeasy^®^ Plus Mini kit (Qiagen, Hilden, Germany) and cDNA was synthetized using the High Capacity cDNA Reverse Transcription kit (Applied Biosystems, Waltham, USA, via Life Technologies) following the manufacturer’s instructions. qPCR was performed on the synthetized cDNA template using the StepOnePlus™Real-Time PCR System (Applied Biosystems). Each PCR reaction contained 20 ng of cDNA, 200 nM of TaqMan assays (Life Technologies), TaqMan Fast Advanced Master Mix (Life Technologies) and ribonuclease-free water to a final volume of 10 µL. Reaction took place as follows: 2 min at 50 °C, 2 min at 95 °C followed by 40 cycles of 1 s at 95 °C, 20 s at 60 °C. Comparative gene analysis was performed with StepOnePlus™ software with β-actin as an endogenous control. Hierarchical clustering and one-way ANOVA with Tukey’s post hoc test were performed on log₁₀-transformed expression data using MetaboAnalyst 6.0 [45]. In heatmaps (Figs. 1A and 3A), colors represent z-scores, indicating each value’s deviation from the row mean in standard deviations. This approach groups genes by expression similarity and provides an overview of experimentally induced transcriptional phenotypes. The following TaqMan assays were used: ACTB - Rn00667869_m1; ARG1 - Rn00691090_m1; IL-1B - Rn00580432_m1; IL-10 - Rn99999012_m1; IL-6 - Rn01410330_m1; NOS2 (iNOS) - Rn00561646_m1; P2RY12 - Rn02133262_s1; SOD2 - Rn00690588_g1; TNF - Rn99999017_m1.

### Immunohistochemistry

Slice cultures were fixed in 4% paraformaldehyde in 0.1 M phosphate buffer for 2 h, incubated in 30% sucrose (AppliChem GmbH, Darmstadt, Germany) and cut into 25 μm sections using a cryostat (CM1850; Leica Biosystems, Nussloch, Germany). Tissue was stained in free-floating sections. Non-specific immunoglobulin reactions were blocked with 5% NGS and slices were permeabilized with 0.3% Triton for 90 min. Sections were incubated with the primary antibody against ionized calcium-binding adaptor molecule 1 (Iba1) (rabbit anti-Iba1, Fujifilm-WAKO Chemicals Europe GmbH, Neuss, Germany) diluted 1:1000 in PBS (pH 6.8) with 10% NGS, 0.3% Triton and 0.1% NaN_3_ overnight. The secondary antibody (Alexa 568 anti-rabbit, Sigma-Aldrich) was diluted 1:1000 in PBS with 5% NGS and 0.3% Triton and applied for 90 min followed by three washing steps (PBS). Sections were then incubated with 4′,6-diamidino-2-phenylindole (DAPI) diluted 1:5000 in distilled water for 5 min and three washing steps (distilled water). The sections were then mounted to microscope slides (SuperFrost Plus, Gerhard Menzel GmbH, Braunschweig, Germany) and dried. 30 µL of fluorescence mounting medium (DAKO, Agilent, Santa Clara, USA) was used for embedding with coverslips. Confocal microscopy images were acquired with a Nikon C2plus confocal microscope by using NIS-Elements software. Image acquisition was performed with a scan size of 2048 pixels and a pin-hole of 1.2 using a Nikon Plan Apo 20x objective.

### Cell counting and morphological analysis

Microglial cells were counted automatically in confocal images from the CA3 region using a macro for the open-source image analysis platform FIJI [46]. Stacks of seven images (0.85 μm steps size) were z-projected and the channels for DAPI and Iba1 separated. For cell counting, a region of interest (ROI) (200 μm × 200 μm) was defined in stratum radiatum, in which neuronal and glial somata are less densely packed. For morphological analysis, edge-based segmentation that defined individual microglial territories permitted the use of larger ROIs (300 μm × 300 μm). FIJI’s subtract background function was employed to reduce noise in the Iba1 image. The DAPI image was converted to binary using FIJI’s “default dark” algorithm for automatic thresholding. The binary DAPI signal was used as a mask on the Iba1 image to clear any signal not colocalizing with DAPI. The remaining Iba1 signal was converted to binary using FIJI’s default dark algorithm. Noise was reduced by the “despeckle” and “remove outlier” functions. Remaining binary shapes were automatically counted by FIJI’s analyze particles function with a cutoff value of 400 pixels to exclude areas where nuclei of other cell types colocalized with microglial ramifications. Microglial cell counts were extrapolated and are given per mm^2^.

Morphological Sholl analysis requires tracing of microglia to obtain binary images of microglial processes (referred to as “skeletons”). This is usually done manually and therefore involves human bias. To reduce this, we developed a FIJI pipeline that minimizes human interaction in pre-processing and automatically traces microglial images. This original method uses watershed segmentation to ascribe an area proportional to soma size to each microglia. The Iba1 signal in these areas was then used for automated tracing and produced individual skeletons of each microglia.

A ROI (300 μm × 300 μm) was defined in z-projected images. The respective channels for DAPI (nuclei marker) and Iba1 (microglial marker) were separated and colocalized. The colocalized areas were converted to a binary image by using FIJI’s „minimum dark“ thresholding algorithm. Then, FIJI’s „find maxima“ function was used to find the centers of colocalized areas, marking the microglia’s nuclei. On the Iba1 image, the „gray scale attribute filtering“ function of the MorphoLibJ-plugin for FIJI was used [47] to enhance areas over 99 pixels in size and thus enhance microglial somata. Then, the image was converted to binary by manual thresholding to include all microglial somata. Somata smaller than 300 pixels were excluded using FIJI’s „analyze particles“ function. Microglial somata were then dilated by 100 pixels and MorphoLibJ’s marker-controlled watershed segmentation was employed to ascribe an area to each microglial cell. On the original Iba1 image, the „unsharp mask“, „despeckle“ and „closing operation“ functions were applied to connect the Iba1-positive areas of microglial processes and remove unspecific signals. Then, pixels outside the segmented areas were cleared. The resulting image was converted to binary using FIJI’s „max entropy“ thresholding algorithm and reconstructed to only include microglia colocalizing with DAPI-stained nuclei. Then, FIJI’s „skeletonize“ function was employed to reduce the microglial shapes to skeletons with 1 pixel diameter. The skeletons were then individually exported and automatically analyzed with FIJI’s “single neurite tracer” plugin [48] using the center of Iba1 and DAPI colocalization as the center for Sholl analysis. All analyses were performed blindly.

### Electrophysiology

**Fig. 2:**
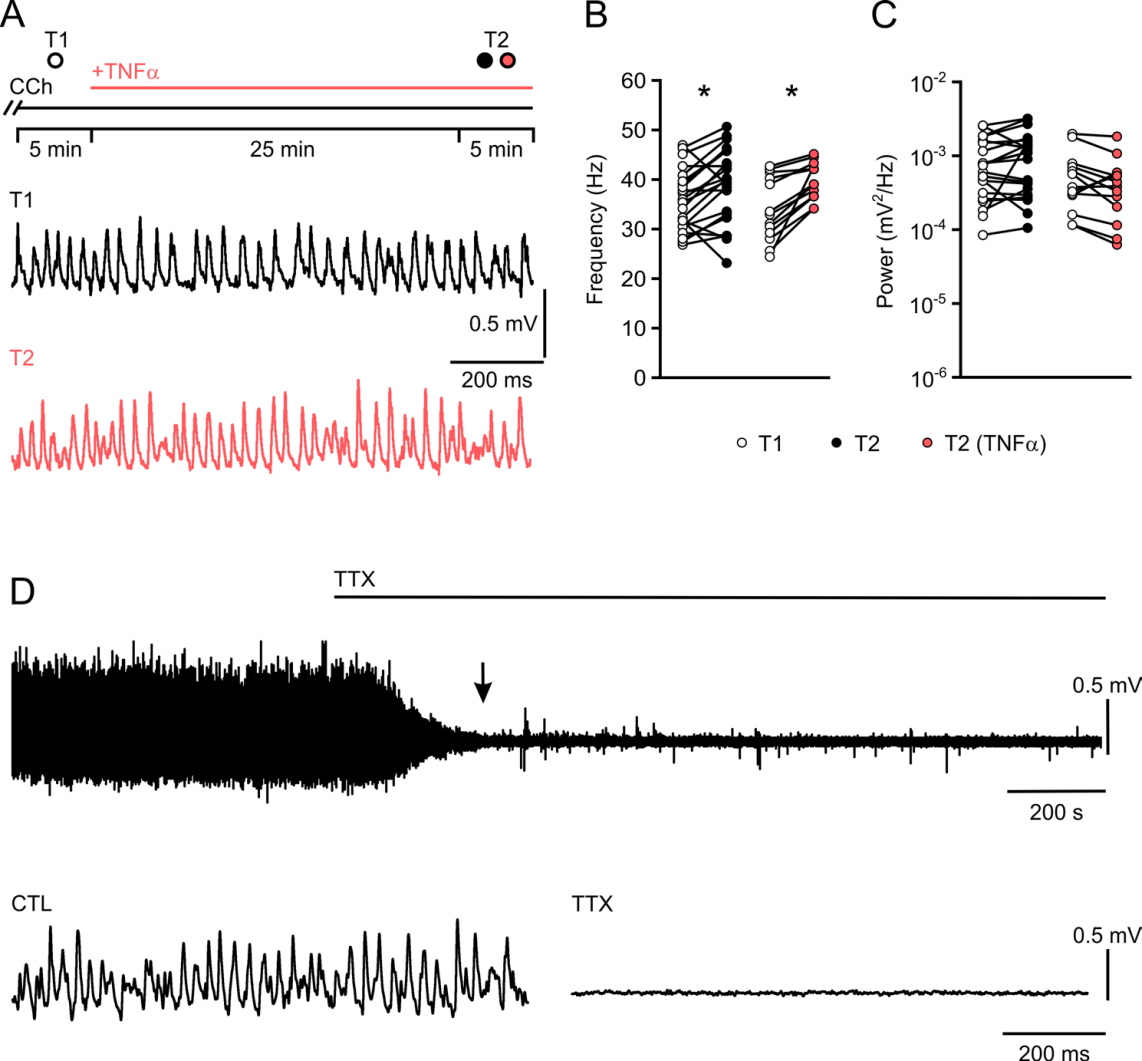
Acute TNF-α does not affect neural gamma oscillations. **A** Gamma oscillations were induced in regular (otherwise untreated) slice cultures by carbachol (CCh, 10 µM, black bar). After 30 min, the first 5 min recording segment was analyzed as reference (T1, open circles in **B** and **C**). The same slices were then acutely exposed to TNF-α (100 ng/mL) through the recording solution (+ TNFα, red bar). After additional 25 min, the second 5 min recording segment was analyzed (T2, red circles in **B** and **C**). Control slices received recording solution without TNF-α (T2, black circles in **B** and **C**). Representative traces of gamma oscillations for CTL (T1, top) and TNFα (T2, bottom). **B** and **C** Peak frequency and peak power of gamma oscillations. Note that the increase in frequency is time-dependent and not specific to acute TNF-α exposure. Comparison by paired t-test (**B**) and Wilcoxon signed-rank test (**C**). *P < 0.008 vs. references (T1). **D** Gamma oscillations were rapidly suppressed within 239 s ± 52 s (black arrow) by the voltage-gated Na^+^ channel blocker TTX (1 µM, black bar), indicating that the use of Biopore^®^ membrane inserts in the interface recording chamber permits rapid tissue saturation with drugs. For n/N slices/animals: (**B**, **C**) 13–22/6–9, (**D**) 15/6

After exposure in the incubator, Biopore^®^ membrane inserts carrying slice cultures were transferred to a custom-built chamber for electrophysiological recordings [26, 49]. In this recording chamber, slice cultures were maintained at the interface between recording solution (artificial cerebrospinal fluid) and the ambient gas mixture. Intact Biopore™ membrane inserts ensure rapid and efficient supply of oxygen, energy substrates and drugs through the recording solution (rate 1.8 mL/min) that flows underneath. The interface condition permits constant oxygen supply (rate 1.5 L/min) from the ambient gas mixture, i.e. 95% O_2_ and 5% CO_2_. The recording solution contained 129 mM NaCl, 3 mM KCl, 1.25 mM NaH_2_PO_4_, 1.8 mM MgSO_4_, 1.6 mM CaCl_2_, 21 mM NaHCO_3_ and 10 mM glucose [26, 36, 37]. The recording solution was saturated with 95% O_2_ and 5% CO_2_, resulting in a pH of 7.3. Recordings were done at 34 ± 1 °C. To induce gamma oscillations (30–70 Hz) in local field potential (LFP) recordings, the cholinergic agonist carbachol (10 µM) was continuously present in the recording solution for about 40 min (except for Fig. 2) and provided robust cholinergic drive in hippocampal tissue [41, 50, 51]. In the experiment on the kinetics of tissue saturation of drugs (Fig. 2D), the voltage-gated Na^+^ channel blocker tetrodotoxin (TTX) (1 µM) was used to suppress action potentials. Standard salts and carbachol were purchased from Sigma-Aldrich, TTX was from American Radiolabeled Chemicals via Biotrend Chemkalien GmbH (Köln, Germany).

The LFP was recorded with microelectrodes (tip resistance of 1–2 MOhm), which were made from GB150F-8P borosilicate glass with filament (Science Products GmbH, Hofheim, Germany) using a Zeitz DMZ Puller (Zeitz-Instruments Vertriebs GmbH, Martinsried, Germany). The electrodes contained a silver-chloride wire and were filled with recording solution. The LFP electrode was positioned in stratum pyramidale of the CA3 region with a mechanical micromanipulator (MX-4, Narishige International Ltd., London, UK). The LFP was recorded with an EXT 10–2 F amplifier in EPMS-07 housing (npi electronic GmbH, Tamm, Germany), low-pass filtered at 3 kHz, and digitized at 10 kHz using CED 1401 interface and Spike2 software [Cambridge Electronic Design (CED), Cambridge, UK] [26, 36, 39]. The LFP was recorded in each slice for about 40 min (except for Fig. 2). Offline data analysis was done in 5 min data segments (minutes 30 to 35 of the recording).

Intracellular recordings were performed in pyramidal cells of the CA3 region using sharp microelectrodes (tip resistance of 70–110 MOhm), which were made from GB150F-10 borosilicate glass with filament (Science Products) using a Flaming/Brown puller P-97 (Sutter Instruments, Novato, CA, USA) [52]. The electrodes contained a silver-chloride wire and were filled with 2 M K-acetate solution. The sharp microelectrode was positioned with a Leitz mechanical micromanipulator (Leica Microsystems GmbH, Wetzlar, Germany). Potentials were amplified using an Axoclamp-2B amplifier (Molecular Devices, San Jose, CA, USA), low-pass filtered at 10 kHz, and digitized at 20 kHz using CED 1401 interface and Spike2 software (CED). Intracellular potentials were recorded in bridge mode and the bridge balance was monitored throughout the experiment. Resting membrane potential was estimated by subtraction of the tip potential following withdrawal from the cell. Input resistance was determined by application of current pulses (-0.2 nA, 200 ms) through the recording electrode and measuring the resulting voltage deflections (at late steady-state level). Duration of positive and negative current injections (range 0.1-1 nA) was controlled using a Master-8 VP stimulator (A.M.P.I., Jerusalem, Israel). Stimulation artifacts were truncated in the illustrated traces. Sharp microelectrode recordings were performed in pyramidal cells that featured stable resting membrane potentials and typical action potential firing [52]. Neurons requiring excessive current injection to maintain resting membrane potentials or showing abnormally high input resistance were excluded.

### Data analysis and statistics

Offline signal analysis of LFPs was performed in MATLAB^®^ 2018b (The MathWorks, Inc., Natick, MA, USA). Power spectral density (power) and peak power frequency (frequency) were determined using scripts developed and kindly supplied by Dr. Jan-Oliver Hollnagel [39, 53]. Data segments of 5 min were processed with a low-pass Butterworth algorithm at 200 Hz corner frequency and processed with Welch’s algorithm with a Hamming window size of 8129 points for calculation of power spectral density, resulting in a bin size of 1.2207 Hz. Network activity was classified as “Gamma” (gamma oscillations) when the peak frequency was higher than 23 Hz at 34 ± 1 °C and the peak power ≥ 10^−4^ mV^2^/Hz [41]. Network activity with lower peak frequency and/or power was classified as „Low activity“. Network activity showing transients with high frequency and amplitude was classified as “Bursts” (neural bursting); the absence of electrical activity was classified as “No activity”. „Bursts“ and „No activity“ were visually identified.

Descriptive analysis of bursts was performed by using the „findpeaks“ function in MATLAB with a prominence cutoff value of 85% to determine the timepoint of individual bursts in the LFP recordings. Continuous wavelet transform using analytical Morlet wavelets was performed, then, the wavelet coefficients at the timepoints determined prior were averaged. For comparison, the same analysis was performed in LFP recordings showing gamma oscillations. A similar approach was used to describe neural high-frequency oscillations during spike-wave discharges in a mouse model of temporal lobe epilepsy [54].

The intrinsic membrane properties of neurons were analyzed using Spike2 software (CED). Amplitudes of the medium afterhyperpolarization were measured as the peak negative voltage deflection following a single action potential evoked during a train of stimuli, compared with the action potential threshold. Amplitudes of the slow afterhyperpolarization were measured as the difference between the resting membrane potential (before stimulation) and the most negative (hyperpolarized) membrane potential reached after the burst of action potentials.

Data derived from “n” cultured slices (or Biopore^®^ membranes or cells) from “N” rat pups. Data from treatments conducted under identical experimental conditions were pooled and are in part repeatedly illustrated in the figures. Individual data points are presented by circles. Unless stated otherwise bar plots represent mean ± SD and box plots represent median and interquartile range, with whiskers indicating minimum and maximum. Statistical significance (P < 0.05) was determined in Graphpad Prism^®^ 11.0 (GraphPad Software, California, USA). Data distribution was tested for normality with the Shapiro-Wilk test. Statistical tests are specified in the figure legends. The Fisher’s exact test compares one type of activity vs. its absence (for example, “Gamma” vs. “No Gamma” oscillations). Figures were created with Graph Pad Prism^®^ (GraphPad Software), and Corel DRAW^®^ X7 (Corel, Ottawa, Ontario, Canada).

## Results

**Fig. 3:**
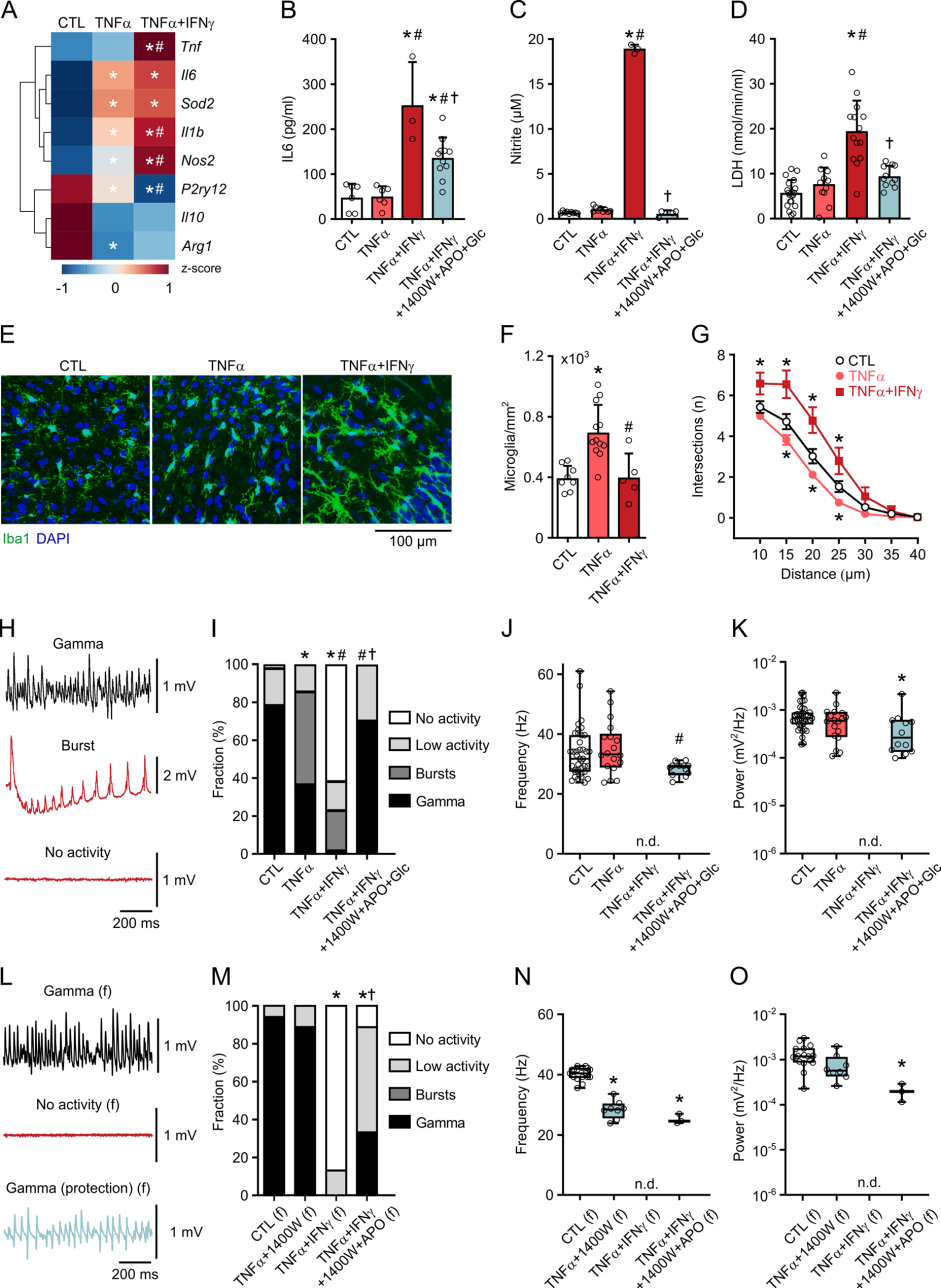
TNF-α plus IFN-γ amplify inflammation and impair neuronal network function. Slice cultures were exposed to TNF-α (100 ng/mL) only or combined with IFN-γ (100 ng/mL) for 72 h. In some experiments, the iNOS inhibitor 1400W (400 µM), the NADPH oxidase inhibitor apocynin (APO, 100 µM) and supplemental glucose (Glc, 6 mM) were additionally applied. Untreated slice cultures served as control (CTL). LFP recordings were made in the presence of carbachol (10 µM). **A** Heat-map depicting alterations in the expression of microglial and inflammatory genes as in Fig. 1A. Comparison by one-way ANOVA with Tukey’s post hoc test. *P < 0.05 vs. CTL and #*P* < 0.05 vs. TNF-α. **B** IL-6 release, **C** nitrite content and **D** LDH activity in the culture medium. Each medium sample originated from three slice cultures. Comparison by one-way ANOVA with Tukey’s post hoc test. (**B**) *P < 0.005 vs. CTL, #*P* < 0.006 vs. TNFα, †*P* < 0.004 vs. TNFα+IFNγ. (**C**) *P < 0.001 vs. CTL, #*P* < 0.001 vs. TNFα, †*P* < 0.001 vs. TNFα+IFNγ. (**D**) *P < 0.001 vs. CTL, #*P* < 0.001 vs. TNFα, †*P* < 0.001 vs. TNFα+IFNγ. **E** Double staining with the macrophage/microglia marker Iba1 and the nuclear marker DAPI. **F** Quantification of Iba1-positive cell somata. Comparison by one-way ANOVA with Tukey’s post hoc test. *P < 0.001 vs. CTL and #*P* < 0.004 vs. TNFα. **G** Automatic Sholl analysis in Iba1-positive cells. Circles represent mean ± SEM. Comparison by two-way ANOVA with Tukey’s post hoc test. *P < 0.03 vs. CTL. The number of intersections reflects the complexity of microglial processes. **H** and **L** Representative LFP traces. **I** and **M** Distribution of network activities. Comparison by Fisher's exact test for Gamma vs. No Gamma. (**I**) *P < 0.001 vs. CTL, #*P* < 0.024 vs. TNFα, †*P* < 0.001 vs. TNFα+IFNγ. (**M**) *P < 0.002 vs. CTL, †*P* < 0.04 vs. TNFα+IFNγ. **J** and **N** Peak frequency and **K** and **O** peak power of slices showing gamma oscillations. Comparison by Kruskal-Wallis test with Dunn’s post hoc test. (**J**) #*P* < 0.03 vs. TNFα. (**K**) *P < 0.007 vs. CTL. (**O**) *P < 0.01 vs. CTL. Comparison by one-way ANOVA with Tukey’s post hoc test. (**N**) *P < 0.001 vs. CTL. (**L**-**O**) from female (f) animals. For n/N membranes/animals: (**A**) 3/3, (**B**) 3-11/3-6, (**C**) 3-9/3-6, (**D**) 11-19/6-11. For n/N slices/animals: (**F**) 5-12/4-6, (**I**-**K**) 17-52/3-21, (**M**-**O**) 9-18/2-5. For n/N cells/animals: (**G**) 24-110/4-6

In this functional neuroimmunology study, we exposed organotypic hippocampal slice cultures from male rats to the proinflammatory cytokines TNF-α and IFN-γ (TNF-α or TNF-α plus IFN-γ) in the incubator and subsequently performed electrophysiological recordings as well as genetic, biochemical and morphological analyses. The cholinergic agonist carbachol (10 µM) was only present in LFP recordings. In selected experiments, we also used slice cultures from female rats to draw more general conclusions (Fig. 3L-O, Supplemental Fig. 2).

### TNF-α induces bursting in neuronal networks

We first explored the molecular effects of slice culture exposure to different TNF-α concentrations (1-1000 ng/mL) for 72 h (“chronic exposure”). These concentrations were shown to induce sufficient activation of TNFR1 in isolated neurons and hippocampal slices [55–57]. Overall, TNF-α induced the upregulation of genes related to inflammation and oxidative stress such as *Il6* (IL-6), *Il1b* (IL-1β), *Nos2* (*iNOS*, inducible NO synthase) and *Sod2* (superoxide dismutase 2), whereas *P2ry12* (purinergic receptor P2Y12) - a marker gene of microglial surveillance - was possibly downregulated (Figs. 1A and 3A).

The changes in gene expression were only partially reflected by IL-6 and NO release (Fig. 1B and C), likely because of the significant dilution in the culture medium (1 mL). The activity of LDH, a general marker of cell death, remained unchanged (Fig. 1D). These findings suggest that chronic TNF-α exposure, even at high concentrations, induces moderate neuroinflammation, including microglial activation.

To investigate the effects of TNF-α on neuronal network activity, we performed LFP recordings after the exposures. Control slice cultures reliably expressed cholinergic gamma oscillations (30–70 Hz), with a peak power at around 35 Hz (Fig. 1E and F) [26, 36]. TNF-α decreased the fraction of slices presenting gamma oscillations. This effect already occurred at 10 ng/mL TNF-α in the medium (with likely much less TNF-α in the slice core). Notably, a significant slice fraction showed recurrent neural bursts that varied considerably in view of amplitude, duration and high frequency components and were classified in bursting types 1 and 2 (Fig. 1E and F, Supplemental Fig. 1). The observed bursting types, however, differed from experimentally evoked epileptiform activity that features “tonic” and “clonic” components lasting for around 50 s in rat slice cultures [58, 59]. Wavelet analysis revealed a prominent peak at 34 Hz during gamma oscillations and two prominent peaks at 0.55 Hz and 100 Hz during bursting type 1 (Fig. 1G). Frequency and power remained regular in slice cultures that still presented gamma oscillations after TNF-α exposure (Fig. 1H and I). TNF-α was reported to induce NO release [22, 24, 60], and moderately elevated NO levels were sufficient to affect neural oscillations in slice cultures [27]. We therefore applied the drug 1400W (400 µM) - a potent and selective inhibitor of iNOS [23] that was also tested in rat slice cultures [26]. Notably, 1400W suppressed neural bursting and widely preserved gamma oscillations (Fig. 3L-O).

Acute exposure to TNF-α (10–25 min) has been reported to exert differential effects on AMPA and GABA receptor trafficking, including excitatory and inhibitory synaptic strengths, and to increase the frequency of action potentials in cortical neurons [8, 57]. We therefore tested the acute effects of TNF-α (100 ng/mL) on gamma oscillations in regular (otherwise untreated) slice cultures (Fig. 2A) making use of rapid drug tissue saturation (Fig. 2D). During exposure to TNF-α (30 min), the properties of gamma oscillations remained regular (Fig. 2B and C). The increase in frequency over time was also present in control conditions and thus unspecific. TNF-α did neither trigger neural bursting nor epileptiform activity (Fig. 2A). This control experiment argued for more lasting cellular adaptations underlying neural bursting (Fig. 1E-G) such as microglial reprogramming (Fig. 1A).

Collectively, these findings suggest that chronic TNF-α exposure impairs gamma oscillations and induces bursting in neuronal networks, widely mediated by iNOS and NO.

### TNF-α plus IFN-γ induce severe neuronal dysfunction

TNF-α is usually present in more complex molecular contexts in the pathogenesis of diseases like epilepsy, multiple sclerosis and Alzheimer’s disease [1, 12, 29]. The key cytokine IFN-γ, for example, can be released by activated lymphocytes in inflamed tissues, and it effectively primes macrophages/microglia resulting in exaggerated immune responses to Toll-like receptor (TLR) ligands [10, 25, 26, 61]. Moreover, IFN-γ is a potent inducer of *Tnf* gene expression in microglia [2, 62]. To further explore the effects of TNF-α in a more complex proinflammatory situation, we exposed slice cultures to TNF-α (100 ng/mL) plus IFN-γ (100 ng/mL) for 72 h (TNFα + IFNγ).

TNFα + IFNγ strongly enhanced both gene expression and release of inflammatory mediators compared with TNF-α only, also expression of *P2ry12* was further downregulated (Fig. 3A-C). The amplified inflammatory response was accompanied by high LDH activity reflecting increased cell death (Fig. 3D). TNFα + IFNγ induced a similar phenotype in female slice cultures (Supplemental Fig. 2).

TNFα + IFNγ also elicited changes in the morphology of microglial cells. Whereas control slice cultures mainly showed ramified and widely non-overlapping microglia, TNF-α decreased microglial ramification and increased microglial cell numbers (Fig. 3E-G, Supplemental Fig. 3), similar as seen in vitro [63]. Interestingly, TNFα + IFNγ induced a hypertrophic morphological phenotype in microglia featuring increased ramification but regular microglial cell numbers (Fig. 3E-G). TNFα + IFNγ thus only partially mimicked the effects of IFN-γ [27]. The morphology induced by TNFα + IFNγ might reflect the “hyper-ramified” phenotype described as a first stage of microglial activation [64].

In LFP recordings, the partial bursting induced by TNF-α only (Fig. 1E-G) markedly progressed to the loss of electrical activity (“No activity”) in the majority of male and female slice cultures exposed to TNF-α plus IFN-γ (Fig. 3H-O). Accordingly, the slice fraction showing gamma oscillations was suppressed (Fig. 3H-O). The type of bursting also changed partially in TNFα + IFNγ (Supplemental Fig. 1). TNFα + IFNγ may cause neuronal dysfunction and cell death by formation of microglial NO, superoxide and peroxynitrite, including metabolic and oxidative stress [21, 65–67]. We therefore applied 1400W (400 µM) in combination with the NADPH oxidase inhibitor apocynin (100 µM) [23, 39] in TNFα + IFNγ in male and female slice cultures. Notably, this pharmacological intervention decreased inflammatory mediators and LDH activity, suppressed neural bursting and preserved gamma oscillations, which was most effective when the glucose concentration in the culture medium was additionally elevated from 4 mM to 10 mM (glucose supplementation) (Fig. 3B-D and H-O).

**Fig. 4:**
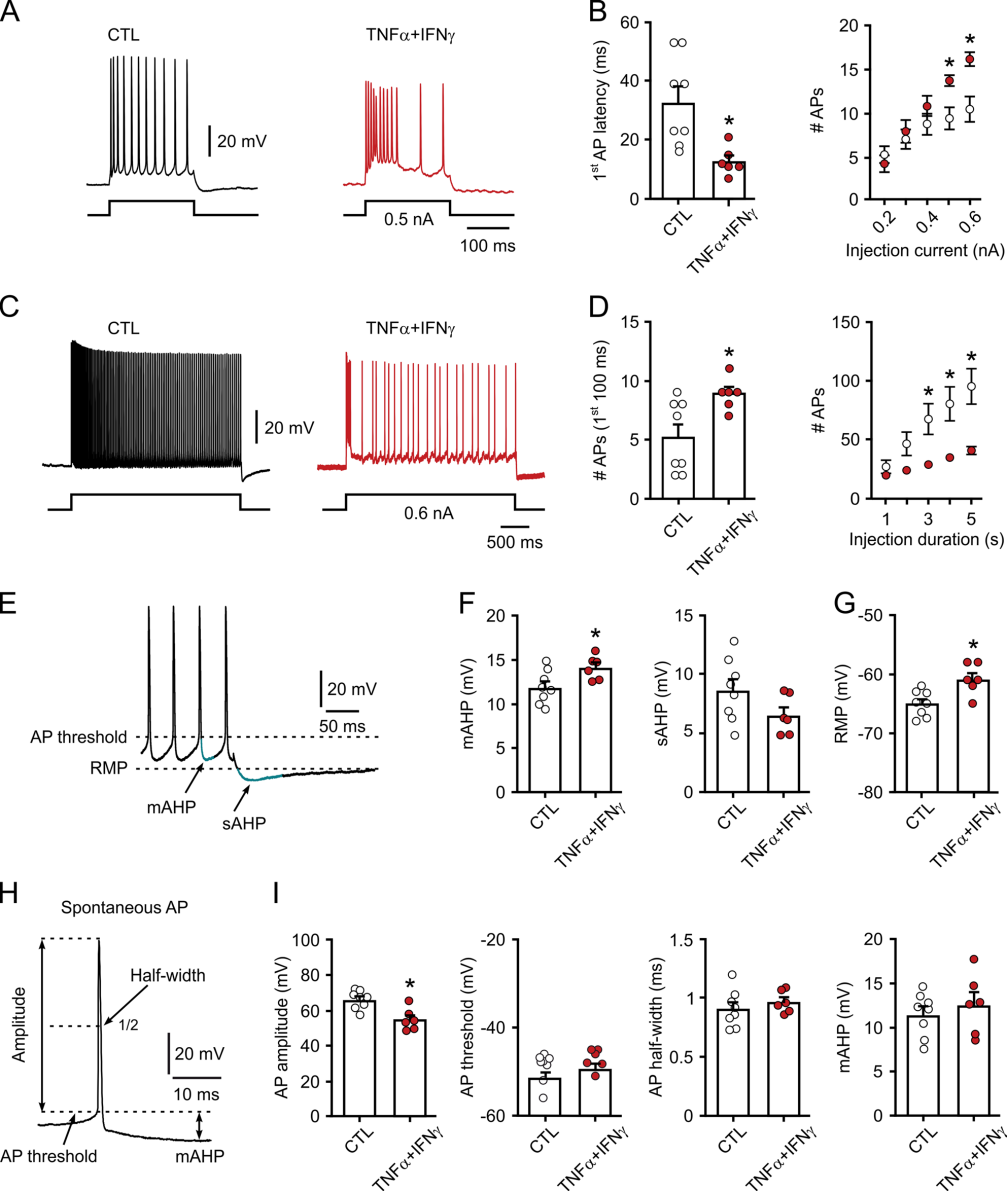
TNF-α plus IFN-γ induce intrinsic hyperexcitability in pyramidal cells. Slice cultures were exposed to TNF-α (100 ng/mL) plus IFN-γ (100 ng/mL) for 24 h. Untreated slice cultures served as control (CTL). Sharp microelectrode recordings were performed to characterize the intrinsic properties of individual pyramidal cells. Data are represented as mean ± SEM. **A** Voltage responses to depolarizing current injection (200 ms, 0.5 nA). **B** Latency of the first action potential (AP) (left) and number of APs (right) in response to increasing steps of current injection (200 ms, 0.2–0.6 nA). Comparison by unpaired t-test with *P < 0.009 vs. CTL (left) and by Sidak’s multiple comparisons test with *P < 0.04 at 0.5 nA and *P < 0.003 at 0.6 nA vs. CTL (right). **C** Voltage responses to prolonged current injection (3 s, 0.6 nA). **D** Number of APs during the first 100 ms of current injection (left) and in response to increasing duration of current injection (0.6 nA) (right). Comparison by unpaired t-test with *P < 0.02 vs. CTL (left) and by Sidak’s multiple comparisons test with *P < 0.05 for 3 s, *P < 0.02 for 4 s, *P < 0.002 for 5 s vs. CTL (right). **E** Voltage response at the late phase of current injection (5 s, 0.6 nA) used for analysis of medium (mAHP) and slow afterhyperpolarization (sAHP). **F** Quantification of amplitudes of mAHP (left) and sAHP (right). Comparison by unpaired t-test with *P < 0.04 vs. CTL. **G** Quantification of resting membrane potential (RMP). Comparison by unpaired t-test with *P < 0.008 vs. CTL. Note that RMP is more depolarized in TNFα + IFNγ. **H** Spontaneously occurring APs were analyzed for various parameters. **I** Quantification of amplitude, threshold, half-width and mAHP (left to right). Comparison by unpaired t-test with *P < 0.003 vs. CTL. For n/N cells/animals: (CTL) 8/6, (TNFα + IFNγ) 6/4

To gain further insight into disturbances of intrinsic neuronal excitability, we performed intracellular electrophysiological recordings in individual CA3 pyramidal cells using sharp microelectrodes thus minimally interfering with the intracellular milieu [52, 68]. Because the phenotype was too severe (Fig. 3H-O) to obtain stable intracellular recordings, exposure of slice cultures to TNF-α (100 ng/mL) plus IFN-γ (100 ng/mL) was shortened to 24 h (Fig. 4). We characterized the intrinsic membrane properties of pyramidal cells by depolarizing current injection at various durations and intensities. TNFα + IFNγ induced the switch from regular spiking to bursting behavior in individual pyramidal cells (Fig. 4A and C), in line with the above findings at the network level. The bursting behavior featured a shorter latency to generation of the first action potential, a higher number of total action potentials during short current injections (Fig. 4B), and a higher number of initial action potentials despite a much lower total number of action potentials during prolonged current injections (Fig. 4D). In addition, TNFα + IFNγ increased the medium afterhyperpolarization (Fig. 4E and F), whereas the resting membrane potential was more depolarized (Fig. 4G). Spontaneous action potentials showed a reduced amplitude (Fig. 4H and I) and occurred occasionally as doublets or triplets (Supplemental Fig. 4). The initial hyperexcitability followed by slowing of spiking during prolonged current injections (intense neuronal activation) is typical for bursting behavior and likely reflects ion channel alterations and metabolic stress in neurons [69, 70].

Collectively, these findings suggest that TNF-α plus IFN-γ induce advanced microglial activation, severe neuronal network dysfunction and neurodegeneration mainly mediated by ROS/RNS and concomitant metabolic and oxidative stress.

### Microglial depletion attenuates neuronal dysfunction and neurodegeneration

**Fig. 5:**
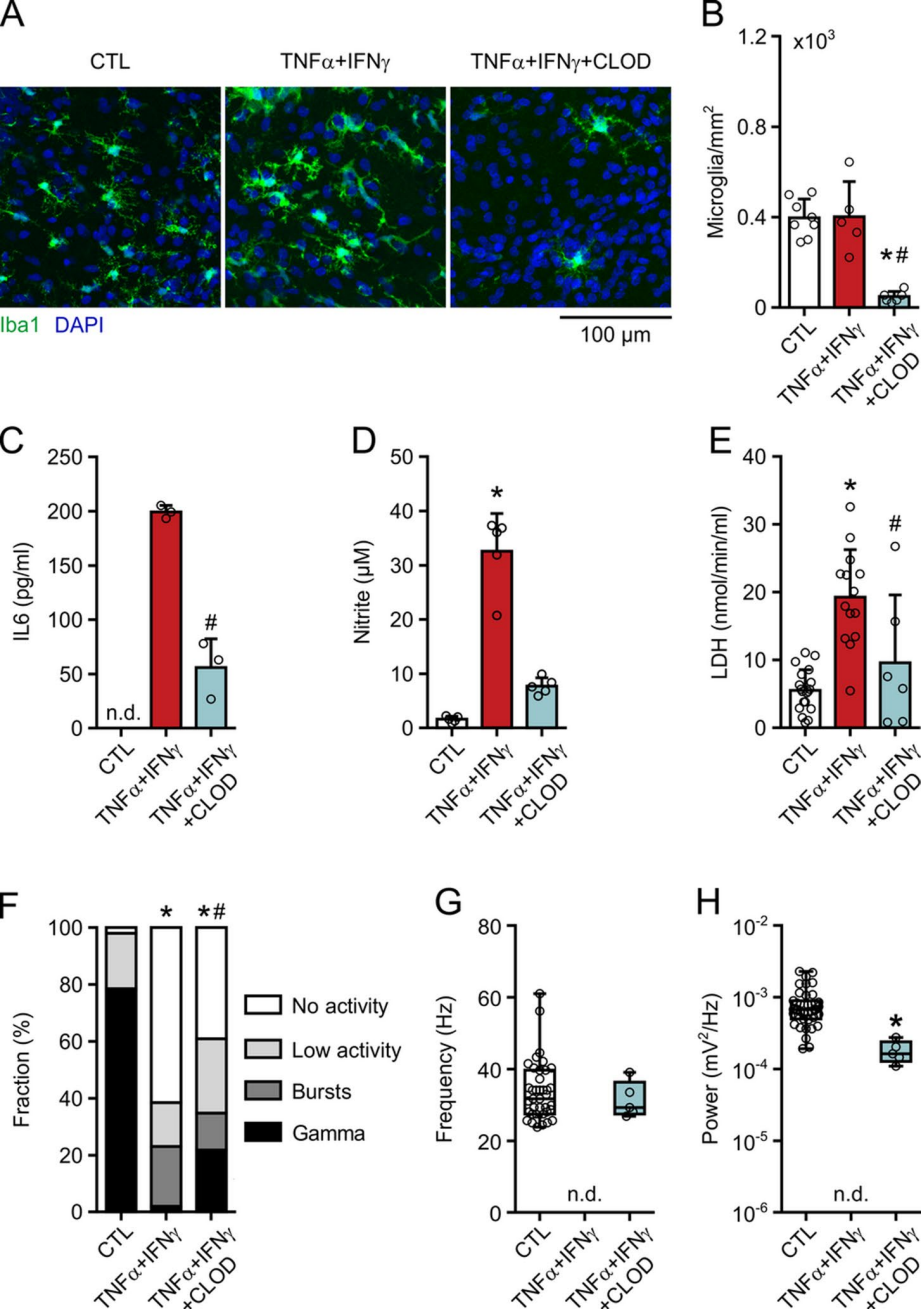
Microglia depletion attenuates neuroinflammation and partially protects gamma oscillations. Slice cultures were exposed to TNF-α (100 ng/mL) plus IFN-γ (100 ng/mL) for 72 h. Microglia were depleted by clodronate (CLOD, 100 µg/mL) from DIV 0 to DIV12. Untreated slice cultures served as control (CTL). LFP recordings were made in the presence of carbachol (10 µM). **A** Double staining with the macrophage/microglia marker Iba1 and the nuclear marker DAPI. **B** Quantification of Iba1-positive cell somata. Comparison by one-way ANOVA with Tukey’s post hoc test. *P < 0.001 vs. CTL and #*P* < 0.001 vs. TNFα + IFNγ. **C** IL-6 release, **D** nitrite content and **E** LDH activity in the culture medium. Each medium sample originated from three slice cultures. Comparison by unpaired t-test (**C**), Kruskal-Wallis test with Dunn’s post hoc test (**D**) and one-way ANOVA with Tukey’s post hoc test (**E**). (**C**) #*P* < 0.001 vs. TNFα + IFNγ. (**D**) *P < 0.002 vs. CTL. (**E**) *P < 0.001 vs. CTL and #*P* < 0.007 vs. TNFα + IFNγ. **F** Distribution of network activities. Comparison by Fisher’s exact test for Gamma vs. No Gamma. *P < 0.001 vs. CTL, #*P* < 0.008 vs. TNFα + IFNγ. **G** Peak frequency and **H** peak power of slices showing gamma oscillations. Comparison by Mann-Whitney test. *P < 0.001 vs. CTL. For n/N membranes/animals: (**C**) 3/3, (**D**) 5/5, (**E**) 6–19/6–11. For n/N slices/animals: (**B**) 5–8/3–4, (**F**-**H**) 23–52/8–21

Severe neuronal network dysfunction and neurodegeneration in TNFα + IFNγ might have been triggered by activated microglia [26, 62, 63]. We therefore depleted the microglial cell population by using liposome-encapsulated clodronate (CLOD). Clodronate (100 µg/mL) efficiently reduces microglial numbers in regular (otherwise untreated) slice cultures by around 95%, without affecting the functional properties of neuronal networks and astrocytic syncytia [36]. Similarly, microglial numbers were lowered by 87% in TNFα + IFNγ + CLOD (Fig. 5A and B, Supplemental Fig. 5). The release of IL-6 and NO was also reduced by about 75% (Fig. 5C and D). The residual IL-6 and NO might result from the fraction of microglia resistant to clodronate and/or reactive astrocytes [9, 61, 71]. Notably, LDH activity was significantly reduced in TNFα + IFNγ + CLOD (Fig. 5E).

In electrophysiology, the fraction of slice cultures showing “No activity” partially decreased and the fraction showing gamma oscillations partially increased in TNFα + IFNγ + CLOD compared with TNFα + IFNγ (Fig. 5F). These gamma oscillations showed regular frequencies, whereas the power was reduced compared with controls (Fig. 5G and H).

These findings indicate that severe neuronal dysfunction and neurodegeneration induced by TNF-α plus IFN-γ are partially mediated by activated microglia.

### Inhibition of TNFR1 signaling protects neural gamma oscillations

**Fig. 6:**
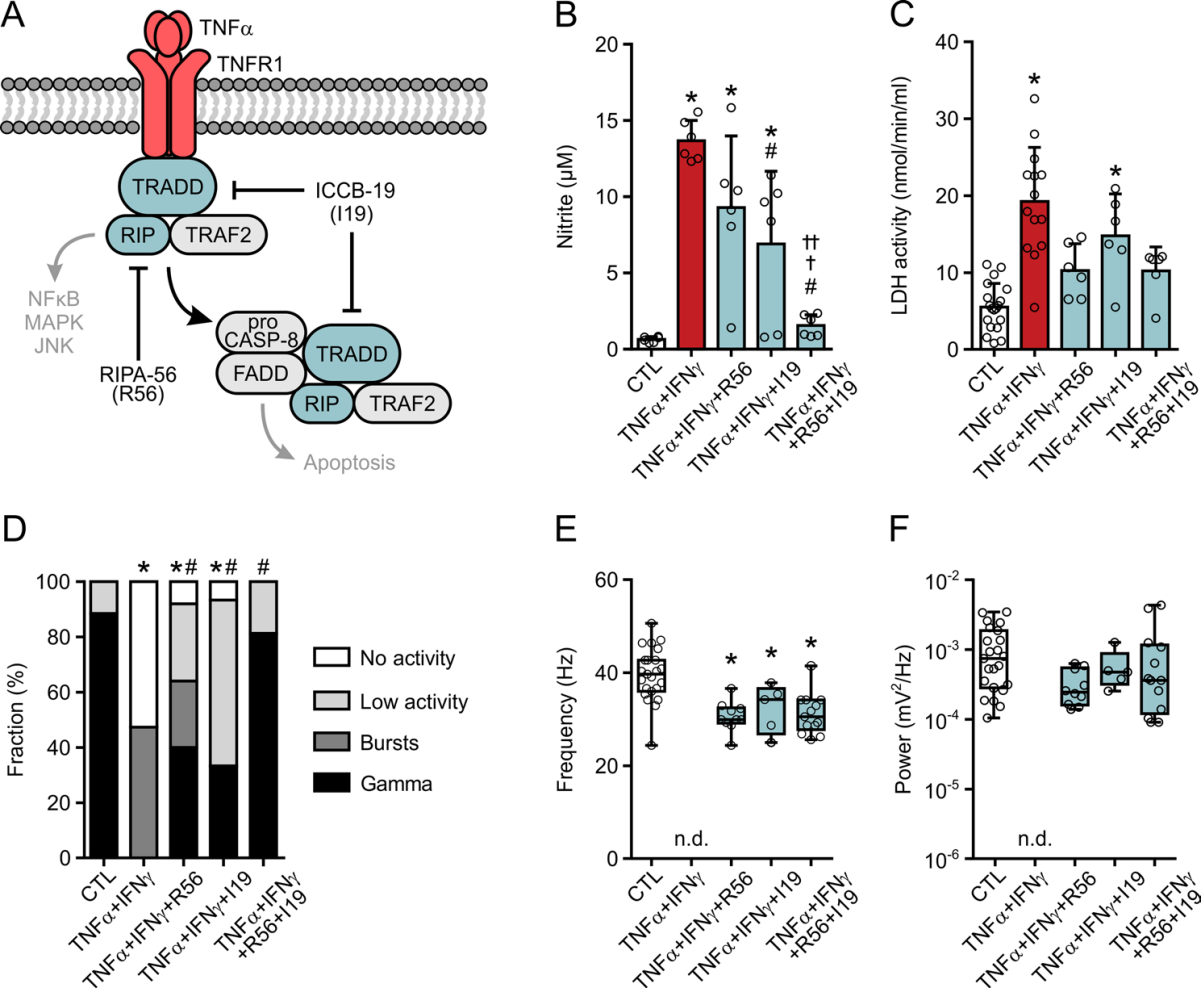
Inhibition of TNFR1 signaling protects gamma oscillations. Slice cultures were exposed to TNF-α (100 ng/mL) plus IFN-γ (100 ng/mL) for 72 h with or without simultaneous exposure to RIPA-56 (R56, 100 µM when alone, 50 µM when combined with I19) and ICCB-19 (I19, 50 µM). Untreated slice cultures served as control (CTL). LFP recordings were made in the presence of carbachol (10 µM). **A** Targets of the small molecule inhibitors RIPA-56 and ICCB-19 are marked by blocking arrows. **B** Nitrite content and **C** LDH activity in the culture medium. Each medium sample originated from three slice cultures. Comparison by one-way ANOVA with Tukey’s post hoc test (**B**) and Kruskal-Wallis test with Dunn’s post hoc test (**C**). (**B**) *P < 0.013 vs. CTL, #*P* < 0.007 vs. TNFα + IFNγ, ✝*P* < 0.002 vs. TNFα + IFNγ + R56 and ✝✝*P* < 0.043 vs. TNFα + IFNγ + I19. (**C**) *P < 0.015 vs. CTL. **D** Distribution of network activities. Comparison by Fisher’s exact test for Gamma vs. No Gamma. *P < 0.001 vs. CTL, #*P* < 0.011 vs. TNFα + IFNγ. **E** Peak frequency and **F** peak power of slices showing gamma oscillations. Comparison by one-way ANOVA with Tukey’s post hoc test (**E**) and Kruskal-Wallis test with Dunn’s post hoc test (**F**). *P < 0.021 vs. CTL. For n/N membranes/animals: (**B**) 6/6, (**C**) 6–19/6–11. For n/N slices/animals: (**D**-**F**) 15–26/4–10

We finally explored the role of TNFR1 in neuronal network dysfunction. TNFR1 is expressed in microglia, neurons and astrocytes, signals through various transduction pathways and mediates inflammatory and pro-apoptotic responses (Fig. 6A), including facilitation of epileptic seizure onset in the brain [12, 72]. We applied pharmacology with the two small molecule inhibitors RIPA-56 (R56) (50 µM or 100 µM) acting on the RIP-1 kinase and ICCB-19 (I19) (50 µM) acting on the TNF-receptor-associated death domain (TRADD) [73, 74].

NO release was lower in slice cultures exposed to TNFα + IFNγ + I19 compared with TNFα + IFNγ, an effect not observed for R56 (Fig. 6B). Notably, NO release and LDH activity returned to control levels when both inhibitors were combined, indicating that inflammation and neurodegeneration depend on TNFR1 signaling in this model (Fig. 6B and C).

In electrophysiology, the fraction of slice cultures showing gamma oscillations was similar compared with controls when RIPA-56 and ICCB-19 were applied in combination (TNFα + IFNγ + R56 + I19) (Fig. 6D). Moreover, the power of gamma oscillations was fully preserved (Fig. 6F). The frequency was somewhat lower but still in the gamma band (30–70 Hz) (Fig. 6E), which might reflect additional mechanisms such as IFN-γ signaling altering the expression of multiple genes [27, 75]. Application of RIPA-56 (TNFα + IFNγ + R56) or ICCB-19 (TNFα + IFNγ + I19) was less effective but still partially suppressed neural bursting and partially preserved gamma oscillations (Fig. 6D-F). Application of R56 + I19 alone did not affect the properties of gamma oscillations in regular (otherwise untreated) slice cultures (Supplemental Fig. 6). Interestingly, RIPA-56 and ICCB-19 also suppressed neural bursting and widely preserved gamma oscillations during TNF-α exposure (TNFα + R56 + I19) in female slice cultures (Supplemental Fig. 2).

These findings suggest that TNFR1 signaling is crucial for TNF-α-induced neuronal dysfunction. Moreover, pharmacological inhibition of TNFR1 signaling is effective not only in preventing neurodegeneration but also in protecting neural gamma oscillations.

## Discussion

The present study was designed to explore the effects of the proinflammatory cytokines TNF-α and IFN-γ on microglial activation, neural network oscillations and intrinsic membrane properties of pyramidal cells, and to test options for pharmacological intervention.

### Functional neuroimmunology in slice cultures

We used rat organotypic hippocampal slice cultures that normally feature well-preserved cytoarchitecture [27, 36]. The resident microglial cells show a widely non-activated phenotype characterized by ramified morphology, expression of surveillance markers such as P2RY12, CX3CR1 and TMEM119, and very low levels of activation markers such as iNOS and major histocompatibility complex class II [26, 34, 36, 50], which we confirm in control slice cultures by a partially different set of markers (Fig. 3). Moreover, the resident microglia are dispensable for neuronal homeostasis and neuromodulation underlying synaptic transmission and gamma oscillations in slice cultures prepared from postnatal rat pups [36]. Notably, different phenotypes of activated microglia can be reliably induced in slice cultures by experimental exposure to TLR ligands such as LPS and peptidoglycan, with distinct changes in microglial surveillance and activation markers [26, 34, 39, 50]. The microglial responses to LPS or IFN-γ in slice cultures are similar to those described in the rodent brain [10, 13]. In brain inflammation, however, the cellular and molecular immunological situation is more complex, often differs depending on the disease and also involves neuroprotective mechanisms such as invasion of regulatory T cells and release of anti-inflammatory IL-4, IL-10 and transforming growth factor β [76–78]. In slice cultures, the lack of blood-derived leukocytes during experimental exposures is advantageous for investigating microglia-neuron interactions in view of neuronal dysfunction and neurodegeneration. The interactions between microglia (innate immunity) and T cells (adaptive immunity) are not covered, however [10, 26, 34, 35, 39].

Slice cultures tolerate culture medium containing 4 mM glucose as well as ambient atmosphere in the incubator (normal air plus 5% CO_2_) [26, 36, 41]. Both partial oxygen pressure and glucose concentration are lower in the slice core where they are near the physiological range [43, 49]. Such glucose levels promote mitochondria content and oxidative phosphorylation in neurons in vitro [79]. These metabolic aspects are important for interpretation of the neuroprotective effect of supplemental glucose (Fig. 3).

We mainly focused on gamma oscillations (30–70 Hz) that show high energy demand and thus provide a suitable readout for neuronal network dysfunction, well before neurodegeneration occurs [10, 27, 67, 80]. Gamma oscillations associate with higher brain functions such as perception, memory and cognition in vivo, and they require timed synaptic transmission between excitatory principal cells and inhibitory interneurons [31, 67, 81].

### Neuronal dysfunction and neurodegeneration induced by TNF-α and IFN-γ

The pleiotropic cytokine TNF-α can be released by activated microglia as well as activated border-associated macrophages, monocytes, T cells and/or natural killer cells infiltrating the brain parenchyma in pathologic conditions [12, 26, 39]. TNF-α also activates microglia and promotes microglial TNF-α release (Figs. 1 and 3) [5, 60]. TNF-α can also enter the brain parenchyma across the blood-brain barrier during systemic inflammation, including sepsis [1–4, 11].

In the brain, TNFR1 is expressed in microglia, astrocytes and neurons, and TNFR1 signaling associates with facilitation of epileptic seizure onset and potentiation of excitotoxicity [7, 8, 12, 17]. TNFR2 expression is more restricted to immune cells but also present in neurons and oligodendrocytes, and it is thought to be protective [2, 72, 82–84].

Chronic TNF-α exposure associated with a moderate proinflammatory microglial phenotype (Figs. 1 and 3), similar as described for intracisternal TNF-α injection in male Wistar rats [20]. Exposure to TNF-α (100 ng/mL) also failed to elicit cell death in HT22 murine hippocampal neuronal cells in vitro [85], contrasting findings from primary mixed neuronal-glial cultures [63]. Acutely elevated systemic TNF-α also had robust effects on brain function without triggering *de novo* neuronal cell death in a mouse model of progressive neurodegeneration [4].

Notably, TNF-α partially induced neural bursting (Fig. 1), and additional microglial priming with IFN-γ resulted in severe neuronal network dysfunction and neurodegeneration reflected by high LDH activity in the culture medium and “No activity” in LFP recordings (Fig. 3). The underlying pathophysiological mechanism likely involves several components: (1) Production of NO and superoxide by activated microglia followed by metabolic and oxidative stress in neurons. Neuronal metabolic stress likely results from increased glucose consumption by proliferating and activated microglia that undergo glycolytic metabolic reprogramming (“metabolic shift”) as well as from inhibition of mitochondrial respiration by NO in neurons [42, 43, 65, 86, 87]. (2) Glutamate release from activated microglia [2, 56]. (3) Enhanced insertion of Ca^2+^-permeable AMPA and/or NMDA receptors in the cell membrane of neurons [8, 88]. Such functional changes carry the risk of neuronal Ca^2+^ overload, enhanced ROS/RNS production, attenuation of glutamate transport in adjacent astrocytes, and neuronal cell death [2, 89, 90]. (4) Induction of apoptosis and necroptosis in neurons, similar to other disease models pairing TNF-α with pathogenic stimuli or drugs [14, 73, 85, 91, 92]. (5) Alterations in astrocyte-neuron signaling resulting in enhanced glutamate release from presynaptic terminals [7, 17, 93]. Elevated levels of extracellular glutamate generally support neuronal hyperexcitability and excitation-inhibition imbalance [12, 94, 95].

Using intracellular recordings with sharp microelectrodes, we found that TNFα + IFNγ induced a more depolarized resting membrane potential (Fig. 4). In addition, depolarizing current injections elicited a brief burst of action potentials followed by slowing of spiking with pronounced afterhyperpolarization. Such alterations in intrinsic membrane properties are typical for burst firing behavior in neurons and likely reflect changes in membrane ion currents (Na^+^ and K^+^ channels) and metabolic stress partially induced by NO and other ROS/RNS [69, 70, 96–99].

Neural bursting on the network level might additionally reflect an excitation-inhibition imbalance caused by increased expression of excitatory glutamatergic AMPA receptors accompanied by enhanced endocytosis of inhibitory GABA_A_ receptors, which can be evoked by TNFR1 activation in hippocampal neurons [8, 55]. Proinflammatory microglia, however, may mediate homeostatic adjustment of excitatory synapses under certain conditions [5].

The amplified microglial immune response in TNFα + IFNγ likely built up neuronal hyperexcitability finally leading to loss of electrical activity and neurodegeneration (Figs. 3 and 4). Notably, we found that inhibition of iNOS and NADPH oxidase plus glucose supplementation suppressed neural bursting and widely preserved gamma oscillations in TNFα + IFNγ + 1400W + APO + Glc (Fig. 3). These findings argue for the concept that TNF-α plus IFN-γ induce the production of NO and other ROS/RNS in microglia and thereby cause metabolic and oxidative stress in neurons [21, 22, 24, 60, 100]. In addition, dysfunction in fast-spiking, inhibitory interneurons may transiently contribute to excitation-inhibition imbalance and boost metabolic and oxidative stress [2, 10, 39, 58, 67]. Glucose supplementation likely enhanced aerobic glycolysis for ATP production and supported the pentose phosphate pathway for oxidative stress handling (NADPH generation for the glutathione pathway) in neurons [100–102]. In this line, the infusion of glucose attenuated acute cognitive impairments in mice treated with LPS and in patients suffering from inflammatory trauma-induced delirium [103]. Although effective in structural and functional neuroprotection, we cannot exclude that 1400W (400 µM) and apocynin (100 µM) also interfered with other cellular targets [104, 105].

Microglial depletion reduced IL-6 and NO release, attenuated neurodegeneration but only partially protected neural gamma oscillations in TNFα + IFNγ + CLOD (Fig. 5). This contrasts somewhat in vitro observations [63] and might reflect more complex regulatory mechanisms in cortical tissue making data interpretation harder. These mechanisms might include (1) ROS/RNS release from the residual microglia with unknown cellular properties that were resistant to clodronate-treatment, (2) reduced availability of the soluble TNFR, which is a potential TNF-α antagonist, (3) diverging changes in AMPA and GABA_A_ receptors and (4) increased glutamate release [5, 7–9].

### Functional neuroprotection by small-molecule inhibitors of TNFR1 signaling

TNFR1 signaling has been reported to contribute to acute clinical symptoms such as cognitive decline and sickness behavior [4, 17, 106]. Moreover, TNFR1 signaling has been implicated in the pathogenesis of various CNS diseases, including epilepsy [72, 84], multiple sclerosis [74, 107, 108] and Alzheimer’s disease [12, 90, 109].

Inhibition of TNFR1 signaling with RIPA-56 and ICCB-19, which inhibit RIP-1 kinase and TRADD, respectively, prevented neurodegeneration and almost completely protected neural gamma oscillations in TNFα + IFNγ + R56 + I19 (Fig. 6). RIPA-56 also blocked the progression of demyelination in an experimental autoimmune encephalomyelitis (EAE) mouse model, likely at the stage of monocyte elevation [74]. In chondrocytes, TRADD mediated TNF-α-induced necroptosis and ICCB-19 suppressed chondrocyte damage and cartilage degeneration by inhibiting TNF-α-TRADD-mediated signaling, including dysregulation of autophagy [110]. ICCB-19 reduced iNOS and Cox2 expression induced by TNF-α in mouse embryonic fibroblasts and attenuated TNF-α release evoked by various TLR ligands and IFN-γ in microglia-like BV-2 cells [73]. The related drug Apt-1 effectively induced autophagy and reduced apoptosis in the hippocampus in a mouse model with pathological tau protein transmission [73].

The present and previous studies suggest that TNFR1 signaling in neurons and glial cells is a promising therapeutic target in neuroinflammation.

## Conclusions

We demonstrate that TNF-α and IFN-γ impair neural oscillations and induce neurodegeneration mainly by a microglial NO mechanism that is sensitive to metabolic support and pharmacological intervention. The functional neuroprotection achieved by blockade of TNFR1 signaling or inhibition of iNOS and NADPH oxidase (plus glucose supplementation) was more effective than microglial ablation only. Future studies are required to dissect the cellular effects of TNFR1 signaling inhibitors in microglia, astrocytes, excitatory and inhibitory neurons, and to explore therapeutic potentials, for example, in sepsis, multiple sclerosis and Alzheimer’s disease in human patients.

## Supplementary Information

Supplementary Material 1.

## Data Availability

All data generated or analyzed during this study are included in this published article and its supplementary information file.

## Abbreviations

APO: Apocynin
CLOD: Clodronate
CNS: Central nervous system
DAPI: 4′,6-diamidino-2-phenylindole
DIV: Day in vitro
EAE: Experimental autoimmune encephalomyelitis
I19: ICCB-19
Iba1: Ionized calcium-binding adaptor molecule 1
IFN-γ: Interferon-γ
IL: Interleukin
iNOS: Inducible nitric oxide synthase
LDH: Lactate dehydrogenase
LFP: Local field potential
LPS: Lipopolysaccharide
NGS: Normal goat serum
NO: Nitric oxide
PBS: Phosphate buffered saline
R56: RIPA-56
ROI: Region of interest
ROS/RNS: Reactive oxygen and nitrogen species
SOD2: Superoxide dismutase 2
TLR: Toll-like receptor
TNF-α: Tumor necrosis factor-α
TNFR: TNF receptor
TRADD: TNF-receptor-associated death domain
TTX: Tetrodotoxin
1400W: *N*-3-aminomethyl-benzyl-acetamidine
