# Supplementary material for: TNF-α and IFN-γ impair neural oscillations and induce neurodegeneration by microglial nitric oxide, metabolic and oxidative stress
Source: J Neuroinflammation. 2026 May 8;23:160. doi: 10.1186/s12974-026-03835-x (PMC13192180; doi:10.1186/s12974-026-03835-x)
Supplement: Supplementary file 1 — Supplementary Material 1. [file 12974_2026_3835_MOESM1_ESM.pdf]

Supplementary Material

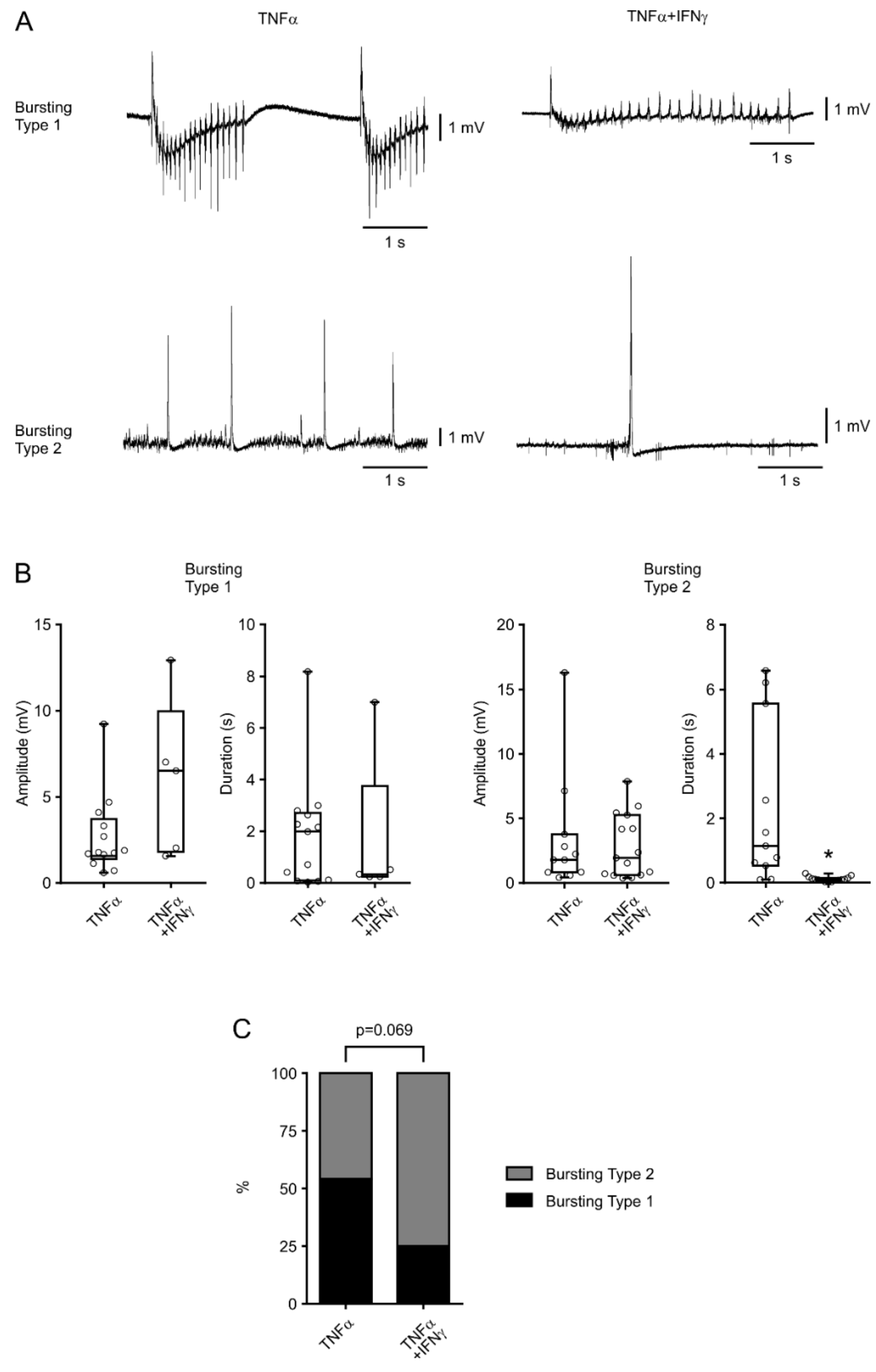

Supplemental Fig. 1. Variable neural bursting in slice cultures exposed to TNF-α and IFN-γ. (A) Representative traces of LFP activity. A large fraction of slices presented recurrent burst activity characterized by an initial large-amplitude transient followed by oscillating population spikes in the range between 7 to 30 Hz accompanied by a negative field potential shift (Bursting Type 1). Another fraction of slices showed shorter bursting transients (Bursting Type 2). (B) Properties of neural bursting was assigned to two categories. Left: Amplitude and duration of Bursting Type 1. Right: Amplitude and duration of Bursting Type 2. Each data point represents 10 averaged burst events per slice. Comparisons with Mann-Whitney test. *$P < 0.001$ vs. TNFα. (C) Distribution of Bursting Types 1 and 2. Bursting phenotypes were confirmed visually in LFP recordings. Comparison with Fisher's exact test. For n/N slices/animals: 5-15/2-12.

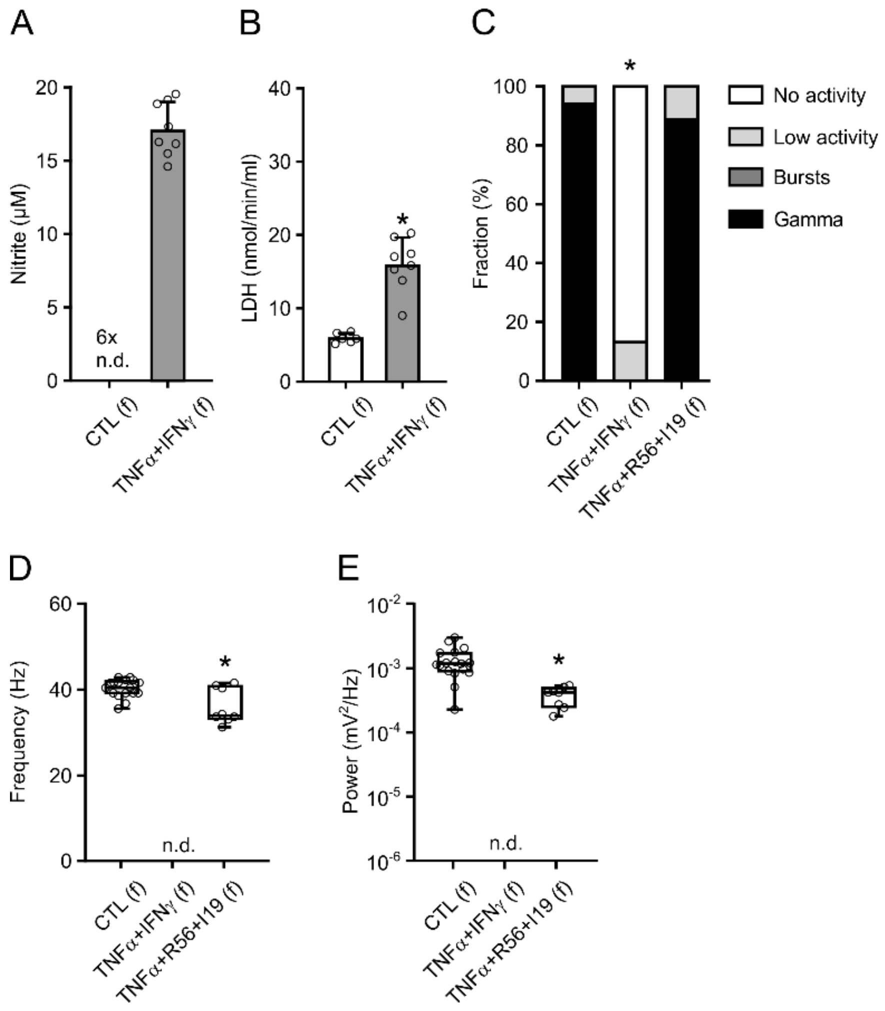

Supplemental Fig. 2. Exposures to TNF-α, IFN-γ and inhibitors in female slice cultures. Slice cultures were prepared from female rats and exposed for 72 h to TNF-α (100 ng/ml) plus IFN-γ (100 ng/ml) or TNF-α (100 ng/ml) in combination with RIPA-56 (R56, 50 µM) plus ICCB-19 (I19, 50 µM). Untreated slice cultures served as control (CTL). LFP recordings were made in the presence of carbachol (10 µM). (A) Nitrite content and (B) LDH activity in the culture medium. Each medium sample originated from three slice cultures. Comparison by unpaired t-test. *P < 0.001 vs. CTL. (C) Distribution of network activities. Comparisons by Fisher's exact test. *P < 0.002 vs. CTL for Gamma vs. No Gamma. Peak frequency (D) and peak power (E) of gamma oscillations. Comparison by unpaired t-test. *P < 0.003 vs. CTL. For n/N membranes/animals: (A) 6-8/4-7, (B) 6-8/4-7. For n/N slices/animals: (C-E) 9-18/2-5.

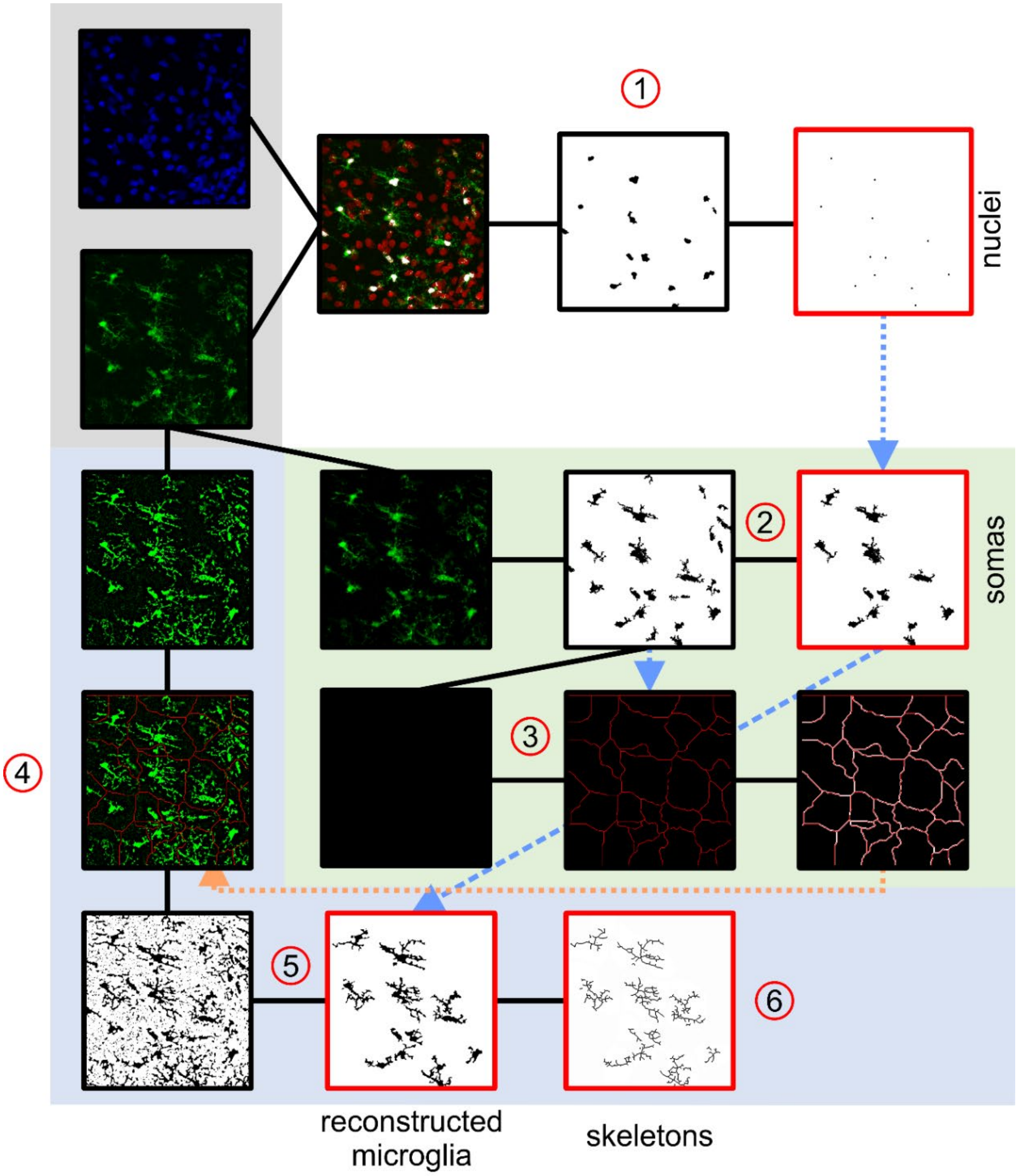
1
nuclei
2
somas
3
4
5
6
reconstructed microglia
skeletons

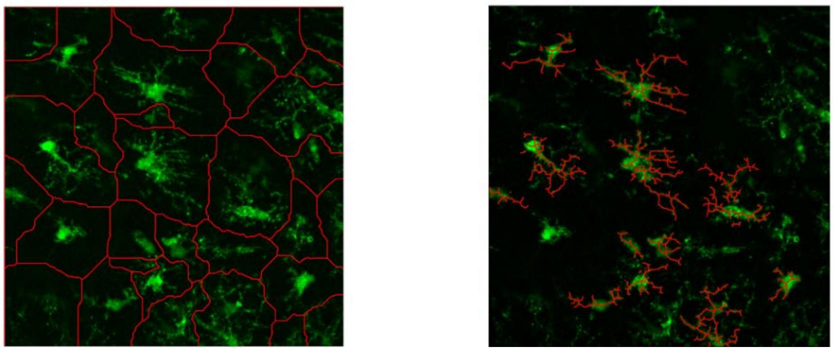

Supplemental Fig. 3. Graphical representation of the morphological analysis. (1) Areas of colocalization between Iba1 and DAPI are automatically thresholded and their maxima used as markers for the positions of the nuclei. (2) The Iba1 image is filtered (gray scale attribute filtering and FIJI's subtract background function), then thresholded. The thresholded image is morphologically recreated by using the nuclei markers, thereby the resulting image only includes Iba1 signal colocalizing with a nucleus. (3) The thresholded image of microglial somata is run through a watershed algorithm using the somata as markers for the entry points, thus creating areas corresponding to each microglial cell. (4) A selection of the watershed areas is used to segment a processed Iba1 image (unsharp mask, radius of 8; despeckle operation; closing operation, radius of 2), which is automatically thresholded. (5) The binary image is reconstructed using nucleus-containing microglial somata as markers. (6) The reconstructed somata are skeletonized. The skeletons are then used for Sholl analysis.

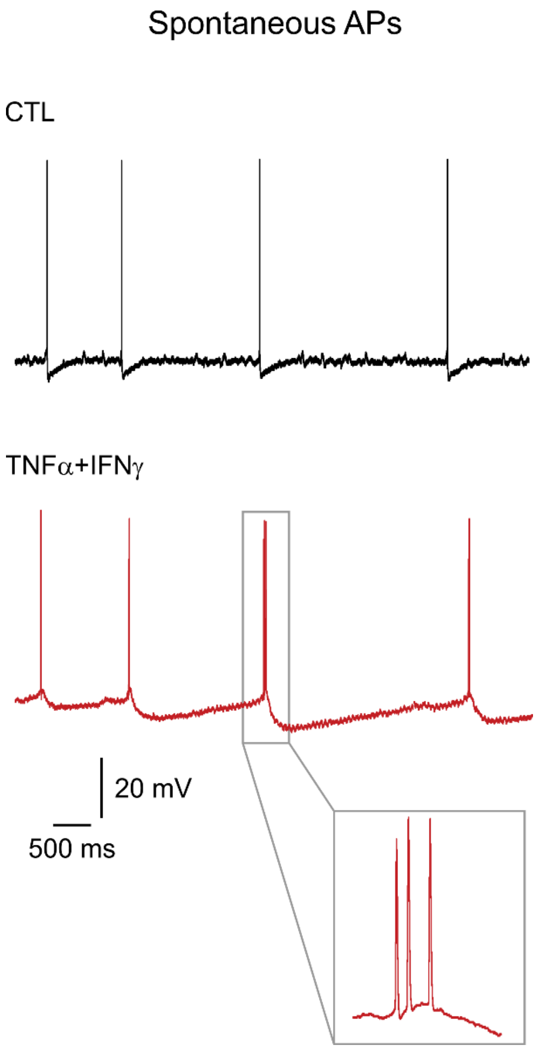

Supplemental Fig. 4. Spontaneous action potentials in CA3 pyramidal cells. Slice cultures were exposed to TNF-α (100 ng/mL) plus IFN-γ (100 ng/mL) for 24 h. Untreated slice cultures served as control (CTL). Intracellular recordings using sharp microelectrodes were performed to characterize the intrinsic properties of individual pyramidal cells. According to the pre-defined exclusion criteria, 1 out of 9 cells (CTL) and 6 out of 12 cells (TNFα+IFNγ) were excluded from analysis (P = 0.1588, Fisher's exact test). Representative traces showing spontaneous action potentials in CTL (black) and in TNFα+IFNγ (red). Half of the neurons in TNFα+IFNγ (3 out of 6 cells) occasionally showed spontaneous spike doublets (2 APs) or triplets (3 APs).

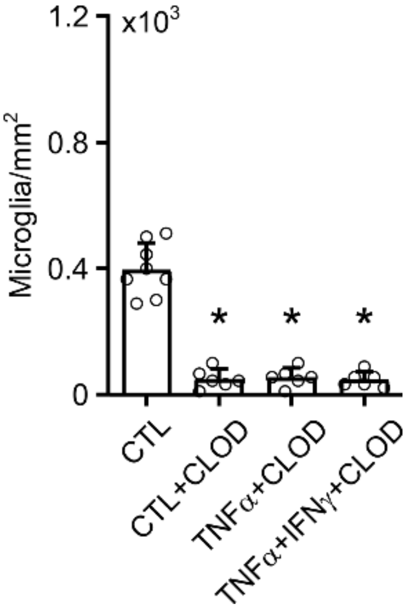

Supplemental Fig. 5. Quantification of Iba1-positive cells. The number of microglial cells was analyzed in stratum radiatum of the CA3 region in untreated slice cultures (CTL) and slice cultures treated with clodronate (CLOD) for microglial ablation with or without TNF-α (100 ng/mL) and IFN-γ (100 ng/mL). Comparison by one-way ANOVA followed by Tukey's post hoc test. *P < 0.001 vs. CTL. For n/N slices/animals: 6-8/3-4.

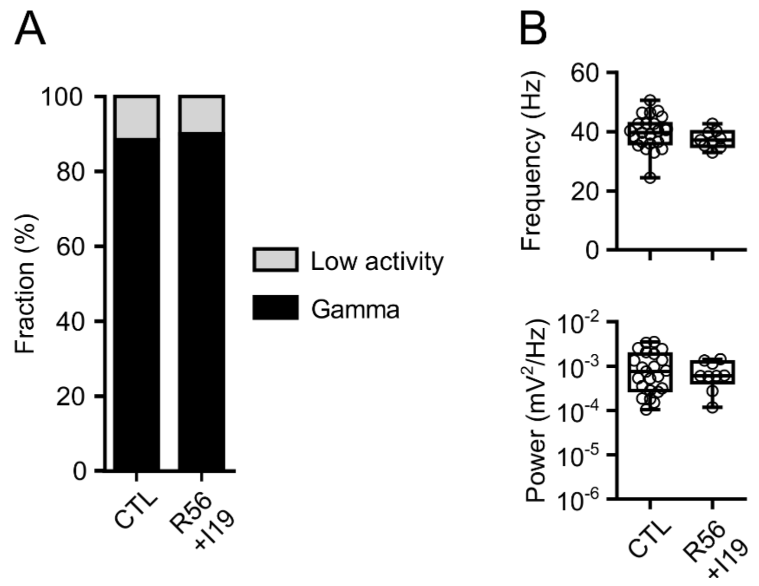

Supplemental Fig. 6. TNFR1 inhibition by RIPA-56 and ICCB-19 in slice cultures. Regular (otherwise untreated) slice cultures were exposed to RIPA-56 (R56, 50 µM) plus ICCB-19 (I19, 50 µM) for 72 h. (A) Distribution of network activities. Comparison by Fisher's exact test (no difference). (B) Gamma oscillation properties: peak frequency (top) and peak power (bottom). Comparisons by unpaired t-test (frequency) and Mann-Whitney test (power) (no differences). For n/N slices/animals: 10-26/4-10.
